# To establish an indirect ELISA method for the detection of human brucellosis based on signal peptide-truncated recombinant Cu/Zn SOD and its preliminary clinical verification

**DOI:** 10.3389/fcimb.2026.1933037

**Published:** 2026-09-08

**Authors:** Chao Wei, Chanjuan Zhang, Meiling Ren, Kaiting Zhang, Huanhuan Yang, Ruiyan Han, Zixuan Dong, Songsong Xie

**Affiliations:** 1 National Health Commission (NHC) Key Laboratory of Prevention and Treatment of Central Asia High Incidence Diseases, The First Affiliated Hospital of Shihezi University, Shihezi, China; 2 Shihezi Center for Disease Control and Prevention, The Eighth Division of Xinjiang Production and Construction Corps, Shihezi, China

**Keywords:** brucellosis, Cu/Zn-superoxide dismutase, indirect ELISA, prokaryotic expression, serological diagnosis

## Abstract

**Background:**

Serological diagnosis of brucellosis is hindered by cross-reactivity with lipopolysaccharide (LPS) antigens, resulting in insufficient specificity and sensitivity, which remains one of the major bottlenecks in current clinical testing for brucellosis.

**Methods:**

To overcome this limitation, we developed an indirect ELISA based on recombinant Cu/Zn superoxide dismutase (rSOD) from Brucella. The N-terminal signal peptide of rSOD was truncated and expressed as a soluble His-tagged protein in Escherichia coli BL21 (DE3) through codon optimization. After purification, detection parameters were optimized using checkerboard titration, and the cutoff value was established using 40 sera from healthy blood donors (mean + 3SD). We evaluated the assay's performance against LPS-ELISA using 106 clinical samples (63 from brucellosis patients and 43 from healthy controls), assessing reproducibility, cross-reactivity with endemic infections (tuberculosis, rickettsiosis, and echinococcosis), and diagnostic accuracy.

**Results:**

Optimal conditions were determined as 1.25 μg/mL antigen concentration, serum dilution of 1:800, and a cutoff OD₄₅₀ of 0.386. Intra-batch and inter-batch coefficients of variation ranged from 0.93% to 4.60% and 1.09% to 12.88%, respectively. Cross-reactivity rates were 15% for tuberculosis, 5% for rickettsiosis, and 0% for echinococcosis. In clinical testing, the rSOD-ELISA demonstrated a sensitivity of 82.5% (52/63, 95% CI 71.4%–89.9%) and a specificity of 90.7% (39/43, 95% CI 78.4%–96.3%), with a false positive rate of 9.3%. Compared with LPS-ELISA, it showed comparable sensitivity (82.5% vs. 80.9%), higher specificity (90.7% vs. 83.7%), a lower false positive rate (9.3% vs. 16.3%), and an overall agreement of 96.2% (Kappa = 0.92, P < 0.001).

**Conclusion:**

The rSOD-ELISA demonstrates superior diagnostic performance through higher specificity and a lower false positive rate compared with conventional LPS-based tests. Although further validation through larger multicenter studies is needed, it represents a promising supplementary serological tool for brucellosis screening in areas with multiple concurrent pathogens.

## 1 Introduction

Brucellosis is a global zoonotic infectious disease caused by *Brucella* species. It is listed as one of the neglected tropical diseases by the World Health Organization, which seriously threatens the development of animal husbandry and public health safety. It has been reported that 2.1 million new cases of brucellosis occur worldwide every year (Laine et al., 2023), and the epidemic has spread in more than 170 countries and regions on five continents (Pappas et al., 2006; Liu et al., 2018), with the disease burden mainly concentrated in agricultural and pastoral areas of developing countries. China is one of the countries with a high burden of brucellosis in the world, and the number of reported cases continues to rank among the top in the world in the past five years. Among them, Xinjiang Uygur Autonomous Region has always been a high incidence area of brucellosis in China, with a reported incidence rate of 23.33/100–000 in the past five years (Yalkun·Mamatimin et al., 2024). The serum positive rate of the occupational population (herdsmen, veterinarians, slaughtering and processing personnel) is as high as 15%-20%. The clinical manifestations of brucellosis are lacking specificity, and the acute stage of brucellosis is mainly characterized by long-term fever, hyperhidrosis, arthralgia, and fatigue, which is highly overlapping with tuberculosis, hepatic echinococcosis, and rickettsial disease, all of which are endemic infectious diseases with high incidence in the Xinjiang region. Most patients in the acute stage progress to chronic brucellosis due to missed diagnosis and misdiagnosis, and have irreversible complications such as joint abnormalities, nervous system damage, and endocarditis. The treatment period is prolonged (Bosilkovski et al., 2019; Qureshi et al., 2023). Due to the lack of laboratory conditions required for bacterial culture in primary medical institutions, and the existing serological methods having many cross-reactions and insufficient accuracy, the development of high-specificity and high-sensitivity diagnostic methods suitable for grassroots promotion has become an urgent need for brucellosis prevention and control in Xinjiang.

At present, the laboratory diagnosis of brucellosis mainly relies on pathogen culture, serological detection, and molecular biological methods (Gusi et al., 2019). Bacterial culture is the “gold standard” for diagnosis, but it is time-consuming, with high biosafety requirements and low sensitivity (Yagupsky et al., 2019; Freire et al., 2024). Serological methods are the main means of clinical diagnosis, among which rose Bengal plate agglutination test (RBPT) and tube agglutination test (SAT) are the most widely used. However, SAT is complicated in operation, highly subjective in result interpretation, and has serious cross-reaction with *Yersinia enterocolitica O:9* and *Vibrio cholerae*, leading to a high false positive rate (Lavigne et al., 2025). Although ELISA still requires standard laboratory equipment (such as microplate readers and incubators) and a controlled environment, due to its high throughput, objectivity, and standardized procedures, it has become the preferred screening technique over other serological methods in primary health care settings. However, the current commercial kits at home and abroad all use lipopolysaccharide (LPS) as the core antigen, and two major defects cannot be ignored: First, there are serious cross-reactions between LPS antigen and *Yersinia enterocolitica O:9*, *Vibrio cholerae*, etc., and the false positive rate is high, which is easy to lead to clinical misdiagnosis and mistreatment. Second, the distribution of *Brucella* LPS antigen was heterogeneous among **Brucella* melitensis*, **Brucella* abortus*, and **Brucella* suis*. Some detection methods based on LPS have been well applied in the diagnosis of bovine brucellosis, but their performance in non-bovine brucellosis may be affected by differences in intermediate antigens (Chart et al., 1992; Kittelberger et al., 1998; Nielsen et al., 2004; Abdoel et al., 2008; Bonfini et al., 2018). While several protein antigens such as *Omp22, BP26, Omp16, Omp25, Omp31, Omp2b, DnaK*, and *Elongation Factor Tu (EF-Tu)* have been utilized in ELISA development or molecular diagnostics, their practical application is limited by low sensitivity, inadequate discriminatory capacity, and cross-reactivity with other endemic diseases (Seco-Mediavilla et al., 2003; Zhao et al., 2019; Bai et al., 2021; Yao et al., 2022; Wu et al., 2023; WuDong et al., 2026; Yun et al., 2026). In view of the inherent defects of the above existing antigens, the development of new protein antigens that are highly conserved among species, have low homology with the host, and can reflect the activity of infection has become the key to breaking through the bottleneck of brucellosis diagnosis. Cu/Zn superoxide dismutase (Cu/Zn SOD), as a key virulence factor of *Brucella* to resist host innate immunity, just has the characteristics of the above ideal antigen (Tian et al., 2020).

Copper-zinc superoxide dismutase (Cu/Zn SOD) is a key virulence factor for *Brucella* to resist the host’s innate immunity. It eliminates the reactive oxygen free radicals produced by macrophage respiratory bursts, enabling the bacteria to survive in the host cell for a long time (Beck et al., 1990; Gee et al., 2005). Previous studies have confirmed that Cu/Zn SOD has strong immunogenicity and can induce the infected host to produce high levels of specific IgG antibodies (He et al., 2002; Oñate et al., 2003; Gee et al., 2005). Notably, as an ideal diagnostic antigen, SOD has high sequence conservation within the *Brucella* genus and has an extremely low homology with the SOD of host mammals. This characteristic significantly reduces the interference of the host’s own immune background and gives it excellent genus-specific detection potential (Beck et al., 1990). One study using recombinant SOD to diagnose brucellosis in animals did not show a significant cross-immune reaction with *Yersinia enterocolitica* O:9 or *Salmonella* (Fu et al., 2025), but all have had limitations such as small sample sizes, expression of the recombinant protein in the form of inclusion bodies, and lack of validation in human serum (Tsogtbaatar et al., 2008). To date, there have been no studies systematically evaluating its application value in human brucellosis diagnosis, nor have standardized ELISA detection methods based on this antigen been established.

Based on the above research background, this study aims to solve the core problems of brucellosis serological diagnosis methods, such as serious cross-reaction, poor applicability among species, and insufficient accuracy in primary diagnosis. Firstly, by cutting out the N-terminal signal peptide of Cu/Zn SOD and optimizing the codon in E. coli, an efficient soluble prokaryotic expression vector was constructed, and the recombinant rSOD protein with high purity and high activity was obtained. Secondly, the reaction parameters of indirect ELISA were systematically optimized by checkerboard titration to establish a standardized detection method. Finally, with bacterial culture combined with SAT as the gold standard, the sensitivity, specificity and repeatability of the method were evaluated using 106 clinically confirmed samples and healthy control samples in the Xinjiang region. This study systematically evaluated the application value of Cu/Zn SOD in the diagnosis of human brucellosis. The established method is expected to provide a new technical method for early screening, diagnosis and treatment monitoring of brucellosis in the Xinjiang region, which has important clinical application and public health value.

## 2 Materials and methods

### 2.1 Collection of clinical serum samples

A total of 146 serum samples were collected from the First Affiliated Hospital of Shihezi University from 2025 to 2026. The procedures followed in this study were approved by the Ethics Committee of the First Affiliated Hospital of Shihezi University (Ethics number: KJ2025-547-01). Written informed consent was obtained from all subjects. Three independent sample cohorts were established for this study. The cut-off value establishment cohort included 40 healthy people with negative tube agglutination test (SAT), no epidemiological contact history of brucellosis, and no brucellosis-related clinical manifestations. The serum was only used for the calculation of the positive cut-off value of rSOD indirect ELISA and was not involved in the subsequent clinical diagnostic efficacy verification. The remaining 106 serum samples were included in the clinical validation cohort, of which 63 patients with brucellosis met the diagnostic criteria of the “Diagnosis and Treatment Plan for Brucellosis of the National Health Commission of the People’s Republic of China in 2023”, and 43 demographically matched healthy controls without a history of brucellosis exposure and SAT negative. In addition, a cross-reaction evaluation cohort was established to evaluate the level of cross-reaction of the method in areas with a high incidence of infectious diseases in Xinjiang. For this cohort, 20 serum samples each from patients with tuberculosis, rickettsiosis, and hepatic echinococcosis were collected from the Department of Infectious Diseases and the corresponding specialized departments at the First Affiliated Hospital of Shihezi University. All these cases were strictly confirmed by the gold standard of etiology (bacterial culture or PCR) or imaging (e.g., CT scan for echinococcosis), following the corresponding national diagnostic guidelines. Those with autoimmune diseases, severe hepatic and renal insufficiency, malignant tumors, use of immunosuppressants within the past 4 weeks, pregnancy or lactation, insufficient serum sample size, and incomplete clinical data were excluded. All serum samples were frozen at -80 °C after isolation to avoid repeated freezing and thawing before testing.

### 2.2 Strains, plasmids, reagents, and instruments

*Escherichia coli* DH5α and BL21 (DE3) competent cells, T4 DNA ligase, and restriction enzymes NdeI and Xho I were purchased from TransGen Biotechnology Co., LTD. (Beijing). The prokaryotic expression vector pET-28a (+) and Ni-NTA pro-harmony column were purchased from Sangon Biotechnology Co., LTD. (Shanghai). Based on the *Brucella* abortus genome sequence (GenBank accession No. KF362132.1), USES the SignalP 5.0 server (https://services.healthtech.dtu.dk/services/SignalP-5.0/), predict a 522 bp Cu/zinc SOD gene signal peptide. The coding region of the signal peptide was truncated to retain the mature peptide coding sequence for intracellular soluble expression in *Escherichia coli*. High-fidelity DNA polymerase was purchased from Takara Bio (Shiga, Japan). BCA protein assay kit and 96-well high-binding polystyrene ELISA microplate were purchased from Shanghai Beyotime Biotechnology Co., LTD. Horseradish peroxidase (HRP) -labeled goat anti-human IgG was purchased from Proteintech Group, Inc. (Rosemont, IL, USA). Other reagents were of analytical grade and purchased from Wuhan Servicebio Bio-Tech Co., LTD. (Wuhan, China). Polymerase chain reaction (PCR) amplification was performed using a Mastercycler Nexus X2 thermal cycler (Eppendorf AG, Hamburg, Germany). Mini-PROTEAN Tetra system and ChemiDoc XRS+ imaging system (Bio-Rad Laboratories, Inc., Hercules, CA, USA) were used. USA) were performed by sodium dodecyl sulfate-polyacrylamide gel electrophoresis (SDS-PAGE) and Western blot. Absorbance values for enzyme-linked immunosorbent assay (ELISA) were measured at 450 nm using a Multiskan FC microplate reader (Thermo Fisher Scientific, Waltham, MA, USA). An ultrasonic cell disruptor (Scientz-IID, Scientz Biotechnology Co., Ltd, Ningbo, China) and a 5424 R high-speed refrigerated centrifuge (Eppendorf AG, Hamburg, China) were used. Germany) for bacterial cell lysis and purification of recombinant proteins.

### 2.3 Construction and verification of recombinant expression plasmids

The truncated mature SOD sequence was optimized for *E. coli* expression and chemically synthesized by Wuhan Genecreate Biotechnology Co., LTD. (Wuhan, China) and cloned into the prokaryotic expression vector pET-28a (+) between the NdeI and XhoI restriction sites. The recombinant plasmid was designed to add a 6×His tag to the N-terminus of the truncated SOD protein for subsequent affinity purification and to carry kanamycin resistance fragments for bacterial screening.

The recombinant plasmid was verified by colony PCR (**Table 1**), double restriction enzyme digestion with NdeI and XhoI, and sequencing to confirm the correct insertion of the target gene, complete truncation of the signal peptide sequence, and absence of base mutations. The validated plasmid was extracted using the Plasmid Purification Kit (Tiangen, China) and transformed into *E. coli* BL21(DE3) competent cells for protein expression.

**Table 1:** Primers and PCR conditions used for colony PCR verification.

Primer name	Sequence	Amplification conditions
F	TAATACGACTCACTATAGGG	nitial denaturation: 95 °C, 8 min (1 cycle); Amplification (30 cycles): 95 °C, 30 s; 55 °C, 30 s; 72 °C, 1 min; Final extension: 72 °C, 10 min (1 cycle); Hold: 4 °C
R	GCTAGTTATTGCTCAGCGG	

### 2.4 Prokaryotic expression and solubility analysis of rSOD

Single positive colonies were sampled, inoculated into LB medium containing 50 μg/mL kanamycin and incubated at 37°C with a shaking bed at 180 rpm until OD_600_ reached 0.6 to 0.8. IPTG was added to the medium at a final concentration of 0.1mM and the culture was induced at 25°C for 12 h on a shaker at 140 rpm.

After induction, bacterial cells were collected by centrifugation at 5000×g for 15 min at 4 °C and rinsed twice with precooled sterile PBS (pH 7.4). The cell precipitate was suspended in lysis buffer (20 mM Tris-HCl, 500 mM NaCl, 5% glycerol, pH 8.0) supplemented with 1 mM DTT, 1mg/mL lysozyme, 10 μg/mL DNase I, and a protein inhibitor mixture without EDTA. Lysis was then performed by sonication (30%, 3 s on, 5 s off, total 30 min) in an ice bath. The lysed bacterial solution was centrifuged at 12000×g for 20 min at 4 °C to separate the soluble supernatant from the insoluble precipitate. The expression level and solubility of rSOD were analyzed by 15% SDS-PAGE.

### 2.5 Purification of rSOD by gravity column chromatography

The soluble supernatant after lysis of the recombinant bacteria was filtered through a 0.22μm sterile filter membrane to remove insoluble particles. A 1 mL Ni-NTA preloaded gravity column was used to equilibrate the chromatography column with 10 times the column volume of binding buffer (20mM Tris-HCl, 500 mM NaCl, 20mM imidazole, 5% glycerol, pH 8.0) at a flow rate of 0.5mL/min. The filtered supernatant was incubated inside the Ni-NTA column at 4 °C for 30 min to ensure adequate binding of the 6× His-tagged rSOD to the Ni-NTA solid phase resin. Then, at a flow rate of 1 drop every 3 seconds, the loading effluent was collected for subsequent detection.

After loading, 15 times the column volume of wash buffer 1 (20mM Tris-HCl, 500 mM NaCl, 20mM imidazole, pH 8.0) and 10 times the column volume of wash buffer 2 (20mM Tris-HCl, 500 mM NaCl, 50 mM imidazole, pH 8.0) were used. pH 8.0) washed the column to remove unspecifically bound miscellaneous proteins.

In order to optimize the elution conditions of the target protein, a pre-experiment was first carried out by using an imidazole concentration gradient elution. The concentration of imidazole in the elution buffer was 100, 150, 200, 250, 300, 400, and 500 mM (the base system was 20 mM Tris-HCl, 500 mM NaCl, 5% glycerol, and the base system was 20 mM Tris-hcl, 500 mM NaCl, and 5% glycerol. pH 8.0), each gradient eluate was collected separately using elution buffer at 5 times the column volume for each imidazole concentration. The results of SDS-PAGE showed that the optimal elution window of rSOD was 250 to 300 mM imidazole concentration.

Based on the optimization results of the pre-experiment, the elution buffer containing 250 mM, 300 mM, 400 mM, and 500 mM imidazole was used for stepwise elution in the formal purification. Five times the column volume of elution buffer was used for each concentration, and the elution fractions with each concentration were collected. All the collected samples were detected by 15% SDS-PAGE, and the results showed that only the 250 mM and 300 mM imidazole elution fractions could show a single target rSOD band without obvious contamination of miscellaneous proteins. The two groups of high-purity target protein eluates were combined for subsequent experiments.

### 2.6 Establishment and optimization of the indirect ELISA method by checkerboard titration

Checkerboard titration was used to optimize the reaction conditions. Purified recombinant Cu/Zn SOD protein was coated onto 96-well microplates at concentrations of 10, 5, 2.5, 1.25, 0.625 μg/mL. The dilutions of serum tested were 1∶100, 1∶200, 1∶400, 1∶800 and 1∶1600. Three coating buffers were evaluated: 0.05 M carbonate-bicarbonate buffer (CBS, pH 9.6), 0.1 M Tris-HCl buffer (pH 8.0) and phosphate buffer (PBS, pH 7.4) and two coating conditions (treatment at 37°C for 2 h and at 4 °C overnight). The blocking medium consisted of commercial blocking buffer, 5% nonfat dry milk, and 4% bovine serum albumin (BSA) for 0.5, 1, 1.5, and 2 h, respectively. The incubation time of primary antibody (patient’s serum) was set at 0.5, 1 and 1.5 hours. Horseradish peroxidase (HRP) labeled goat anti-human IgG secondary antibodies were diluted at 1∶2,000, 1∶4,000, 1∶8,000 and 1∶10000, and incubated for 0.5, 1 and 1.5 hours. Three positive controls (sera from patients with confirmed brucellosis) and three negative controls (sera from patients without brucellosis) for each condition were included. The highest positive/negative (P/N) ratio was selected as the optimal condition.

### 2.7 Validation of indirect ELISA methodology

#### 2.7.1 Determination of cutoff value

The ELISA method was used to detect the serum samples of 40 healthy people who were negative for brucellosis by tube agglutination test (SAT), blood smear microscopy and bacterial isolation and culture, and the OD_450_ value was measured. Shapiro-Wilk normality test showed that the data were normally distributed (W = 0.9617, P = 0.191). The mean (x) and standard deviation (SD) were calculated from the OD_450_ values, and the cut-off formula was (x+3SD, which was taken from the mean and standard deviation to calculate the cut-off value. In the detection of clinical samples, if OD_450_≥(x+3SD), the sample was judged as positive; OD_450_≤(x+2SD) was judged as negative, and between the two was suspicious.

#### 2.7.2 Specificity verification

Twenty serum samples from patients with common infectious diseases (including tuberculosis, echinococcosis and rickettsiosis) diagnosed clinically in the Xinjiang region were selected. All the samples were detected by the best ELISA conditions optimized in this study. The test results were determined according to the determined diagnostic cut-off value, and the specificity of the method was calculated. If the above non-brucellosis serum test results were all negative and there was no cross-reaction, it indicated that the method had good specificity and could effectively distinguish brucellosis from other common infectious diseases in the Xinjiang region.

#### 2.7.3 Sensitivity verification

To evaluate the analytical sensitivity, one representative confirmed positive serum sample (confirmed by bacterial culture and SAT) was serially diluted two-fold from 1:50 to 1:6400. Each diluted sample was then tested using the optimal ELISA conditions established above. The highest dilution that still yielded a positive result was recorded as the detection limit of the method.

#### 2.7.4 Repeatability verification

Three brucellosis-positive sera with high (OD_450_:1.5-2.5), medium (OD_450_:0.5-1.5) and low (OD_450_:0.39-0.5) titers and three negative sera were selected as the primary antibodies. The within-run tests were repeated on the same batch of microplate with three replicates per sample, and the within-run coefficient of variation was calculated. The inter-assay repeatability test was carried out on three different batches of microplate with three replicates per sample, and the inter-assay coefficient of variation was calculated.

### 2.8 Detection of clinical samples

A total of 106 serum samples were collected from the Department of Infectious Diseases, the First Affiliated Hospital of Shihezi University, from 2025 to 2026, including 63 brucellosis patients diagnosed by bacterial culture combined with tube agglutination test (SAT) and 43 healthy controls. Three technical replicates were set for each sample to be tested. The results were interpreted according to the established criteria.

### 2.9 Comparison with the LPS-ELISA kit

The control reagent used for comparison was a commercially available LPS-coated human *Brucella* IgG antibody ELISA kit (Catalog No. JL46158D, Jonln Biotechnology Co., Ltd., Shanghai, China). Based on the principle of indirect ELISA, this kit detects serum-specific IgG antibodies and is currently the mainstream reagent for brucellosis serum screening. According to the operating instructions, 106 clinical serum samples (63 confirmed brucellosis patients and 43 healthy people) were detected in parallel, and each sample was set up with three technical replicates.

### 2.10 Statistical analysis

SPSS 26.0 and GraphPad Prism 9.0 were used for data analysis and mapping. Continuous variables were judged to be normal by the Shapiro-Wilk test and were described as mean ± standard deviation when they conformed to normal distribution. ELISA positive threshold to negative serum OD_450_ mean, between $\bar{\text{x}}$+2SD and $\bar{\text{x}}$ + 3SD between sentences is suspicious. The sensitivity, specificity, positive predictive value (PPV), negative predictive value (NPV), and 95% confidence interval (CI) were calculated using clinical diagnosis as the gold standard. The diagnostic efficacy of the two ELISA methods was compared by the McNemar paired chi-square test (continuity correction), and the consistency was evaluated by Cohen’s Kappa coefficient. The formula used was κ = (Po - Pe)/(1 - Pe), where Po represents the observed agreement and Pe represents the expected agreement based on chance. The detailed cross-tabulation and calculations of the Kappa value are provided in **Supplementary Data S1**. The repeatability was expressed as the coefficient of variation (CV), and the intra-assay CV ≤ 10% and inter-assay CV ≤ 15% were considered qualified. Pearson chi-square test was used to compare the positive rate between the two groups, and the odds ratio (OR) and 95% confidence interval (CI) were calculated. All tests were two-sided, and the significance level was α=0.05.

## 3 Results

### 3.1 Transformation of recombinant expression plasmids and verification of positive clones

**Figure 1:**
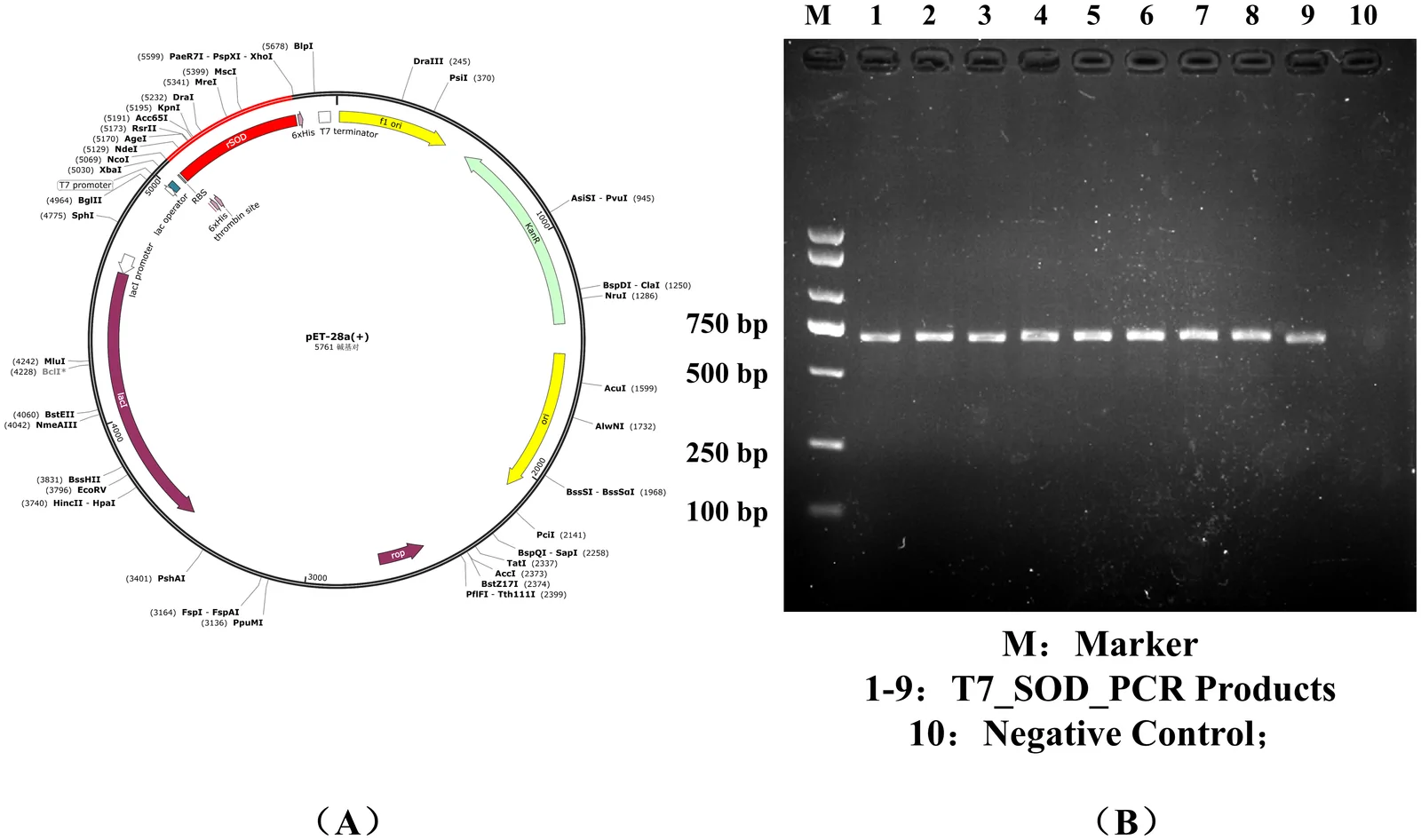
Construction and validation of the signal peptide-truncated recombinant expression plasmid pET-28a-SOD. **(A)** Schematic representation of the recombinant plasmid pET-28a-SOD, illustrating the inserted SOD gene (without the signal peptide) downstream of the T7 promoter, along with the 6×His tag and kanamycin resistance gene.; **(B)** Agarose gel electrophoresis of colony PCR products using T7 universal primers (**Table 1**). Lane M: DNA marker; Lanes 1–9: PCR products from nine randomly selected positive transformants; Lane 10: Negative control. The specific bands indicate the successful transformation of the recombinant plasmid into *E. coli BL21*(DE3).

The recombinant plasmid Pet-28a-signal peptide-SOD (**Figure 1A**) was transformed into *E. coli* BL21(DE3) competent cells and coated on LB solid plates containing 50 μg/mL kanamycin. After 16 h of incubation at 37°C, clear and uniform single colonies grew on the plates.

Random pick 9 single colonies; colony PCR identification using T7 has universal primers (**Table 1**). The results of agarose gel electrophoresis showed that specific bands were amplified in all nine clones (**Figure 1B**), indicating that the recombinant plasmids had been successfully transformed into *Escherichia coli* and could be used for subsequent induction of expression experiments.

### 3.2 Optimization of induced expression conditions and validation of specificity of recombinant SOD

#### 3.2.1 Analysis of recombinant SOD induced expression

**Figure 2:**
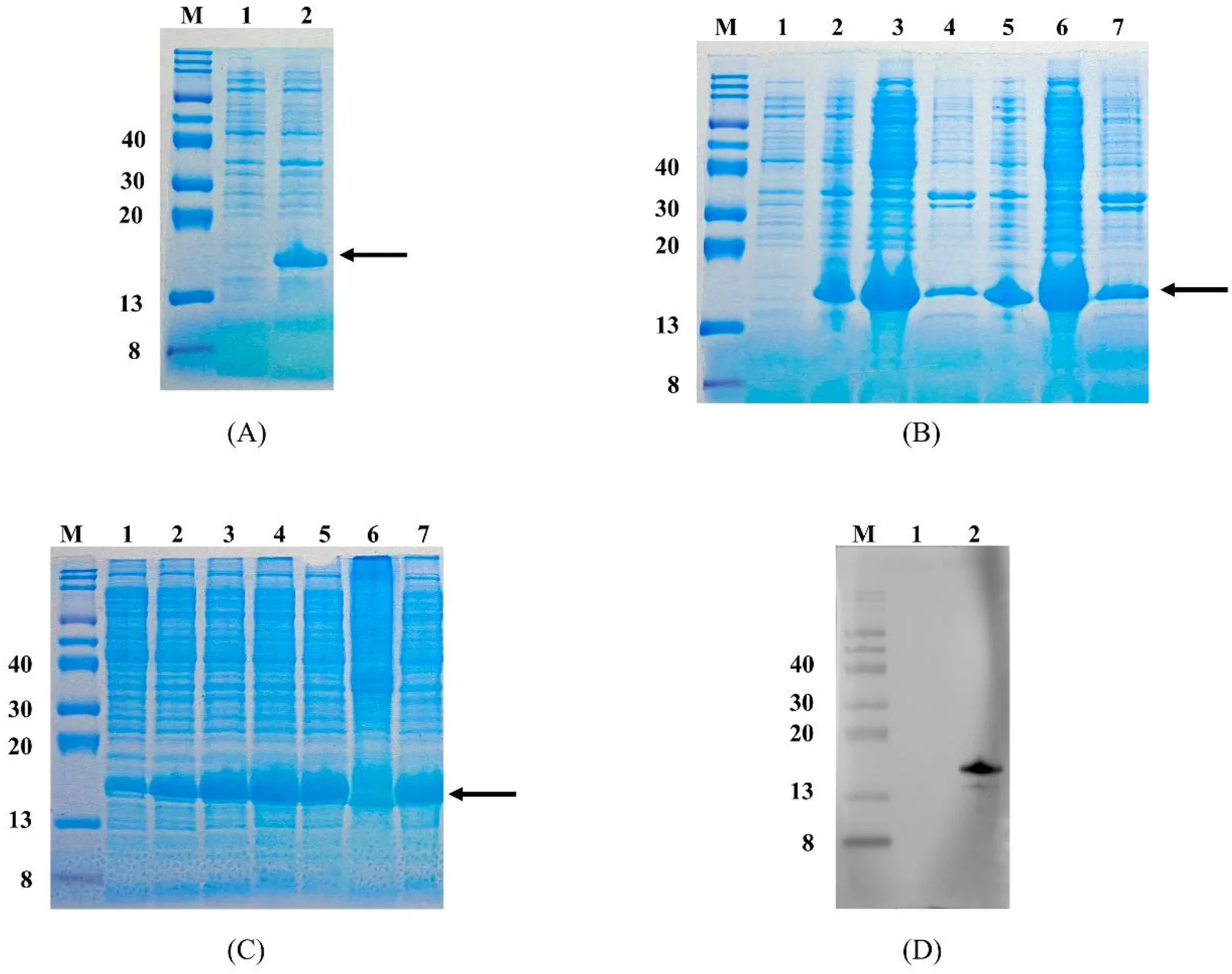
SDS-PAGE and Western blot analysis of the induced expression and optimization of recombinant SOD. **(A)** Expression of rSOD analyzed by 15% SDS-PAGE. Lane M: protein molecular weight marker; Lane 1: uninduced whole-cell lysate; Lane 2: IPTG-induced whole-cell lysate. An obvious protein band at approximately 18.3 kDa was observed in Lane 2. **(B)** Effect of induction temperature on the solubility of rSOD. Lane M: protein marker; Lanes 1 and 4: uninduced and induced whole-cell lysates at 37 °C; Lanes 2 and 3: supernatant and precipitate fractions at 37 °C; Lanes 5 and 6: uninduced and induced whole-cell lysates at 25 °C; Lane 7: supernatant fraction at 25 °C. Low-temperature induction (25 °C) significantly improved the soluble expression of rSOD. **(C)** Effect of IPTG concentration on rSOD expression. Lanes 1 to 7 represent induction with IPTG concentrations of 0.01, 0.02, 0.05, 0.1, 0.2, 0.5, and 1.0 mM, respectively. Maximum expression was achieved at 0.1 mM IPTG. **(D)** Western blot validation of rSOD using an anti-His tag monoclonal antibody. Lane 1: uninduced cell lysate; Lane 2: induced cell lysate. A single specific band at 18.36 kDa confirmed the successful expression of the His-tagged rSOD.

The positive clones were inoculated into LB liquid medium and cultured to OD_600_=0.6 at 37°C. Then 0.5mM IPTG was added and induced at 25°C for 12 h. 15% SDS-PAGE results showed that an obvious specific protein band at about 18.3 kDa appeared in the induced whole bacterial sample, which was consistent with the theoretical molecular weight of recombinant SOD, while no corresponding band was found in the non-induced control group (**Figure 2A**), indicating that recombinant SOD was successfully expressed in *E. coli* BL21(DE3).

#### 3.2.2 Effect of induction temperature on soluble expression

Soluble expression of rSOD was analyzed by induction with 0.5 mM IPTG at 37°C and 25°C, respectively. The results showed that most of the target proteins were distributed in the soluble supernatant and the soluble expression was significantly increased when the low temperature was induced at 25°C (**Figure 2B**). Therefore, 25°C was chosen as the optimal induction temperature.

#### 3.2.3 Effect of IPTG concentration on expression

The transformed cells were induced with 0.01, 0.02, 0.05, 0.1, 0.2, 0.5, and 1.0 mM IPTG for 12 h at 25°C. SDS-PAGE results showed that the expression of the target protein gradually increased with the increase in IPTG concentration. When the IPTG concentration reached 0.1mM, the expression reached the peak, and the expression did not significantly increase with the increase of IPTG concentration (**Figure 2C**). Therefore, 0.1mM was chosen as the optimal IPTG induction concentration.

#### 3.2.4 Specificity verification by Western blot

Pre/post induction samples were taken for Western Blot analysis using anti-His tag monoclonal antibody as the primary antibody. The results showed only a single specific band at 18.36 kDa (**Figure 2D**), confirming that the expressed protein was a His-tagged recombinant SOD.

### 3.3 Purification of recombinant SOD by Ni-NTA gravity column

**Figure 3:**
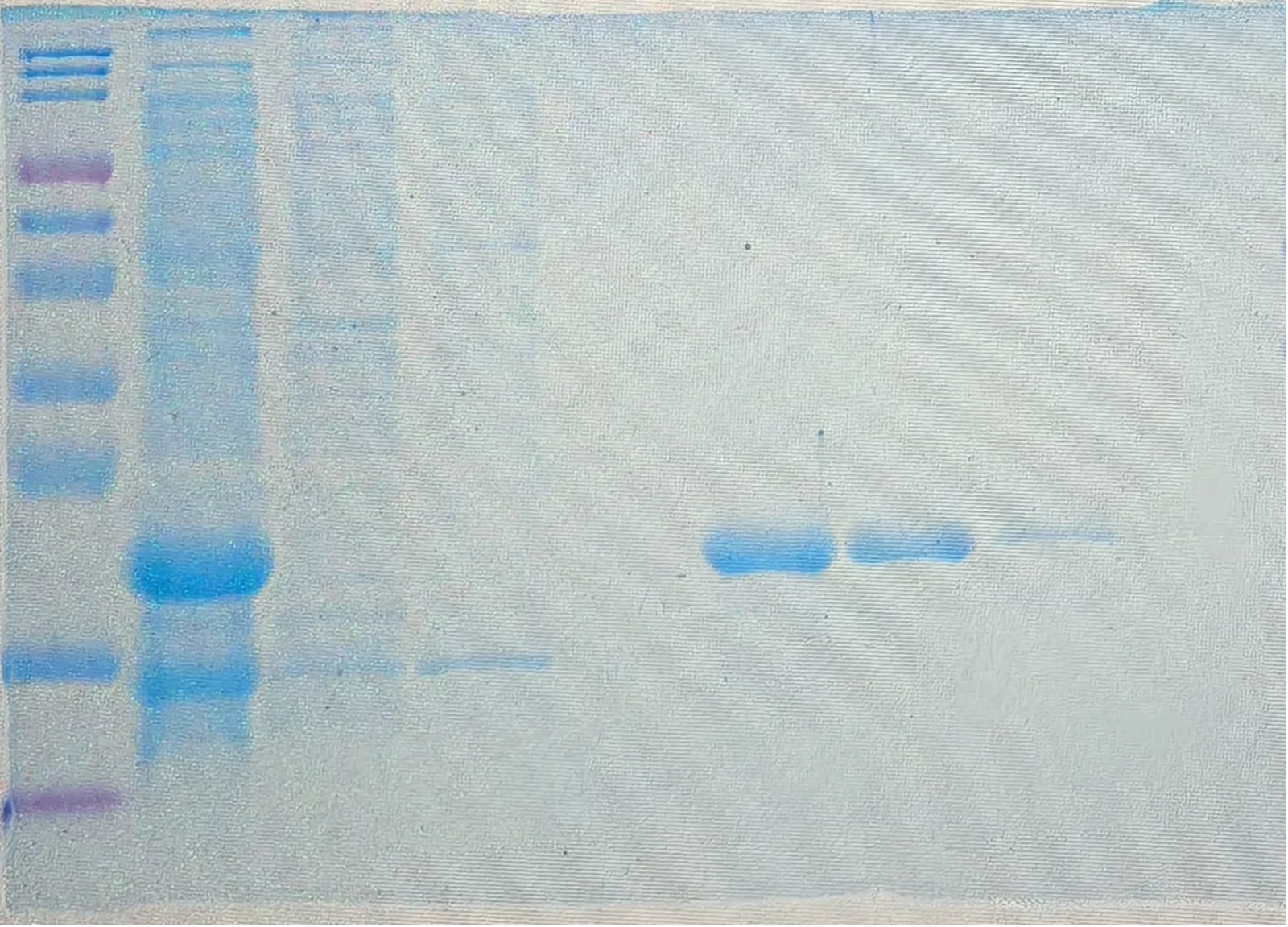
Purification of recombinant SOD via Ni-NTA gravity column chromatography. Lane M: protein molecular weight marker (8–195 kDa); Lane 1: crude bacterial lysate supernatant; Lane 2: flow-through fraction; Lane 3: wash fraction with 20 mM imidazole; Lane 4: wash fraction with 50 mM imidazole; Lanes 5 to 8: elution fractions with 250, 300, 400, and 500 mM imidazole, respectively. The target protein was eluted mainly in the 250 and 300 mM imidazole fractions with high purity.

The recombinant SOD was affinity purified using a Ni-NTA gravity preloading column. After loading, the supernatant was washed with 20 mM imidazole and 50 mM imidazole in order to remove non-specifically bound miscellaneous proteins, followed by gradient elution with 100 to 500mM imidazole. The results of SDS-PAGE (**Figure 3**) showed that the target protein was eluted mainly at the concentration of 250–300 mM imidazole, and the purity of the fraction eluted with 250mM imidazole was the highest. The eluted fractions with higher purity were combined, desalted and concentrated using Millipore ultrafiltration concentration tubes (with a molecular weight cutoff of 10 kDa), and the buffer was replaced with sterile PBS (pH 7.4). The concentration of the concentrated protein was determined to be 11.3mg/mL by the BCA method, and subsequently diluted to 1mg/mL in sterile PBS (pH 7.4) containing 10% (v/v) glycerol, packaged and stored at −80°C.

### 3.4 Establishment and optimization of indirect ELISA detection method

#### 3.4.1 Optimization of the best reaction conditions

**Figure 4:**
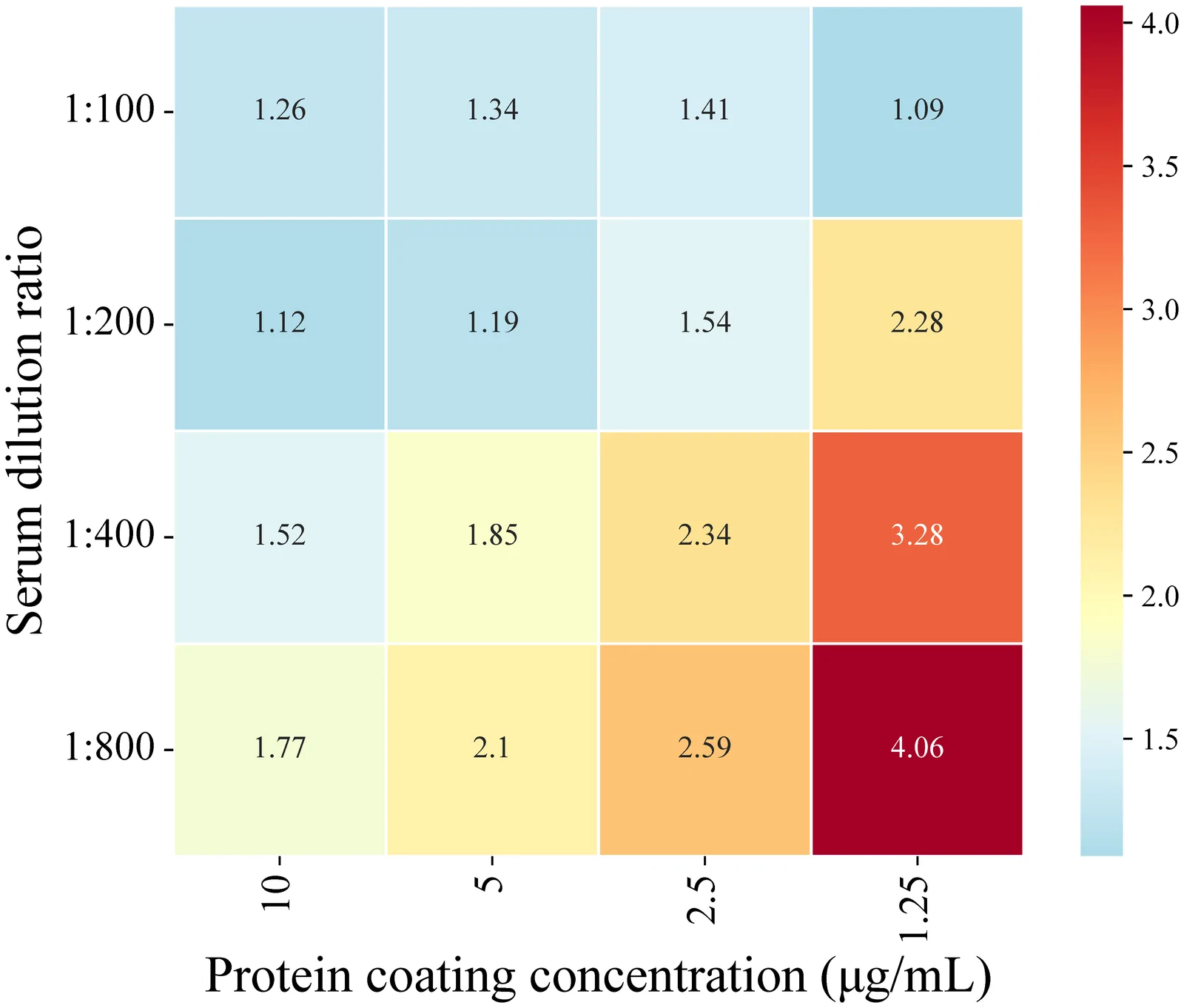
Optimization of the antigen coating concentration and serum dilution for rSOD indirect ELISA using checkerboard titration. The heatmap displays the positive-to-negative (P/N) OD_450_ ratios under varying antigen coating concentrations (10, 5, 2.5, 1.25 μg/mL) and serum dilution ratios (1:100 to 1:800). The highest P/N ratio (4.06) was achieved with an antigen concentration of 1.25 μg/mL and a serum dilution of 1:800.

The optimal antigen coating concentration and serum dilution ratio were determined using a checkerboard titration assay. In this approach, different concentrations of purified rSOD antigen (10, 5, 2.5, 1.25, and 0.625 μg/mL) were coated onto the microplates and reacted with serial dilutions of both positive and negative sera (ranging from 1:100 to 1:1600). The optimal combination was selected based on the highest positive-to-negative (P/N) OD_450_ ratio to maximize the discrimination between positive and negative samples. As shown in the heatmap in **Figure 4**, the highest P/N ratio (4.06) was achieved with an antigen coating concentration of 1.25 μg/mL and a serum dilution of 1:800. Therefore, these specific parameters were selected for all subsequent ELISA procedures. (**Figure 4**).

#### 3.4.2 Determination of antigen coating conditions

After coating the microplate with 1.25 μg/mL antigen, the coating conditions were optimized. The results showed that coating with 0.01 mol/L PBS at 37°C for 2 h had the highest OD_450_ value and a significantly higher P/N value than the other two groups (**Table 2**). Therefore, PBS + 37°C for 2 h was selected as the optimal coating condition.

**Table 2:** Determination of optimal antigen coating conditions.

Coating buffer	Parameter	4°C overnight	37°C 2h
CBS	**P**	2.84	2.85
	**N**	1.14	0.60
	**P/N**	2.48	4.79
Tris	**P**	3.13	3.16
	**N**	0.96	0.77
	**P/N**	3.26	4.13
PBS	**P**	3.02	3.09
	**N**	1.14	0.52
	**P/N**	2.66	5.91^*^

P, OD_450_ of positive serum; N, negative serum OD_450_; P/N, the ratio of positive and negative serum OD values.

*Bold values: The maximum P/N ratio within each parameter group, expressed as the overall best condition for the final scheme selection.

#### 3.4.3 Determination of blocking conditions and blocking time

At 37°C, using commercial rapid blocking solution, 4% bovine serum albumin (4% BSA), and 5% skim milk powder as blocking solution, and blocking for 30min, 60min, 90min, and 120min, checkerboard titration experiments were performed. According to the P/N value, the P/N value of 5% skim milk powder blocking for 90 min was higher than that of the other groups, so 5% skim milk powder blocking for 90 min was selected as the blocking solution (as shown in **Table 3**).

**Table 3:** Determination of closure conditions and closure time.

Blocking reagent	Parameter	30 min	60 min	90 min	120 min
5% skim milk powder	**P**	2.40	1.86	2.14	2.06
	**N**	0.56	0.48	0.38	0.59
	**P/N**	4.27	3.90	5.63^*^	3.47
commercial rapid blocking buffer	**P**	1.39	0.81	0.99	1.12
	**N**	0.60	0.50	0.57	0.61
	**P/N**	2.33	1.63	1.74	1.84
4% BSA	**P**	1.73	1.49	1.66	1.76
	**N**	1.25	1.23	1.47	1.42
	**P/N**	1.38	1.21	1.13	1.24

P, OD_450_ of positive serum; N, negative serum OD_450_; P/N, the ratio of positive and negative serum OD values.

*Bold values: The maximum P/N ratio within each parameter group, expressed as the overall best condition for the final scheme selection.

#### 3.4.4 Optimal reaction time of primary antiserum

To determine the optimal incubation time for the primary antibody (patient serum), the serum was diluted at 1:800 (the optimal dilution determined in Section 3.4.1) and incubated at 37 °C for 30, 60, and 90 min, respectively. The OD_450_ values of both positive and negative sera were measured, and the positive-to-negative (P/N) ratios were calculated to assess the discriminatory power. As shown in **Table 4**, the highest P/N ratio (4.07) was achieved at an incubation time of 60 min. Therefore, an incubation period of 60 min at 37 °C was selected as the optimal condition for the primary antibody in the subsequent ELISA procedures.

**Table 4:** Optimal serum reaction time.

Parameter	30 min	60 min	90 min
P	0.92	1.36	1.47
N	0.24	0.33	0.37
P/N	3.81	4.07^*^	3.98

P, OD_450_ of positive serum; N, negative serum OD_450_; P/N, the ratio of positive and negative serum OD values.

*Bold values: The maximum P/N ratio within each parameter group, expressed as the overall best condition for the final scheme selection.

#### 3.4.5 Determination of dilution and action time of enzyme-labeled secondary antibody

At 37°C, the enzyme-labeled secondary antibody was diluted in 4 dilution ratios of 1:2000, 1:4000,1:8000, and 1:10000, and the P/N values under different conditions were calculated (see **Table 5**). The P/N value of the 1:10000 dilution group was the largest for 30 min, so the optimal dilution and the optimal action time of the enzyme-labeled secondary antibody were 1:10000 and 30 min, respectively.

**Table 5:** Determination of dilution and action time of enzyme-labeled secondary antibody.

Secondary antibody dilution	Parameter	30 min	60 min	90 min
1:2000	**P**	3.32	3.35	3.36
	**N**	1.31	1.61	1.83
	**P/N**	2.54	2.08	1.83
1:4000	**P**	2.58	2.97	3.18
	**N**	0.83	1.16	1.28
	**P/N**	3.10	2.57	2.49
1:8000	**P**	1.51	2.05	2.29
	**N**	0.46	0.66	0.80
	**P/N**	3.30	3.09	2.85
1:10000	**P**	0.96	1.34	1.56
	**N**	0.28	0.43	0.52
	**P/N**	3.38^*^	3.12	3.00

P, OD_450_ of positive serum; N, negative serum OD_450_; P/N, the ratio of positive and negative serum OD values.

*Bold values: The maximum P/N ratio within each parameter group, expressed as the overall best condition for the final scheme selection.

#### 3.4.6 Summary of optimized ELISA conditions

Based on the stepwise optimization described above, the optimal conditions for each step of the rSOD indirect ELISA were determined. The finalized protocol parameters are systematically summarized in **Table 6** for quick reference and easy reproducibility.

**Table 6:** Summary of the optimized indirect ELISA protocol.

Step	Optimized condition(s)	Determined parameters
Antigen coating	Coating buffer/Temperature/Time	PBS (pH 7.4)/37 °C/2 h
	Antigen concentration	1.25 μg/mL
Blocking	Blocking reagent/Time	5% skim milk powder/90 min
Primary antibody	Serum dilution/Incubation time	1:800/60 min
Secondary antibody	HRP-conjugate dilution/Incubation time	1:10,000/30 min
Substrate development	TMB incubation time	15 min (in the dark)
Reaction termination	Stop solution/Measurement	50 μL/well, OD_450_ reading

### 3.5 Validation of indirect ELISA methodology

#### 3.5.1 Determination of the cutoff value

**Figure 5:**
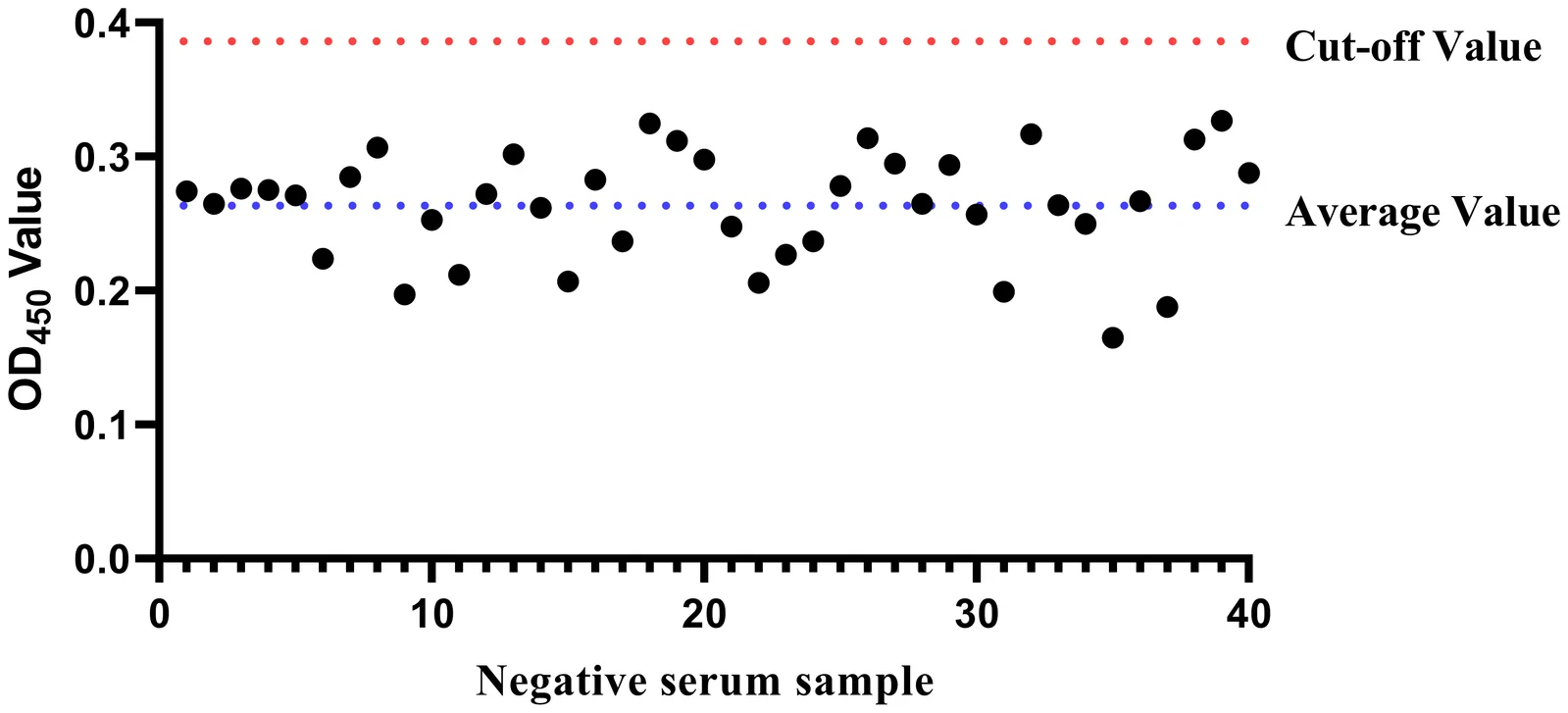
Distribution of OD_450_ values from 40 independent negative serum samples used to determine the cut-off value. The blue dashed line represents the mean OD_450_ value (0.2634), and the red dotted line represents the cut-off value calculated as the mean + 3 standard deviations (SD), which was 0.386. Samples with an OD_450_ > 0.386 were considered positive.

Forty negative serum samples from healthy people were tested by the established indirect ELISA method of rSOD, which was independent of the clinical validation set. The OD_450_ value of each sample was recorded and statistically analyzed. The Shapiro-Wilk normality test showed that the OD_450_ value of 40 negative serum samples conformed to the normal distribution (W = 0.9617, P = 0.191). x =0.2634, standard deviation (SD) =0.0407, (x+3SD = 0.386, (x+2SD = 0.345). That is, samples with an OD_450_ value >0.386 were judged as positive and OD_450_ values <0.345 were judged as negative, and samples with an OD_450_ value between 0.345 and 0.386 were judged as suspicious. (**Figure 5**).

#### 3.5.2 Specificity experiments

To evaluate the specificity of the established rSOD-ELISA, we tested serum samples from patients with clinically prevalent infectious diseases in Xinjiang, China.

Three diseases—tuberculosis, rickettsiosis, and hepatic echinococcosis (cystic echinococcosis caused by *Echinococcus granulosus*)—were selected for specificity evaluation based on three rationales. On the one hand, a recent study by Fu et al. (2025) experimentally demonstrated that the recombinant superoxide dismutase (rSOD) protein shows no cross-reactivity with *Yersinia enterocolitica*, *Salmonella* spp. and other related pathogens, consistent with previously reported findings (Fu et al., 2025). Second, at our local clinical setting, confirmed serum specimens from patients with *Yersinia enterocolitica* or *Vibrio cholerae* infection were extremely scarce, so sufficient samples could not be obtained for systematic validation. Most importantly, the clinical manifestations of brucellosis in Xinjiang, including fever, diaphoresis, and arthralgia, are most often confused with the above three endemic diseases, all of which are highly prevalent in the region. Accordingly, verifying the specificity of the rSOD-based ELISA against these co-circulating infectious diseases carries far greater clinical value for local differential diagnosis and practical application than testing cross-reactivity with rare pathogens.

**Figure 6:**
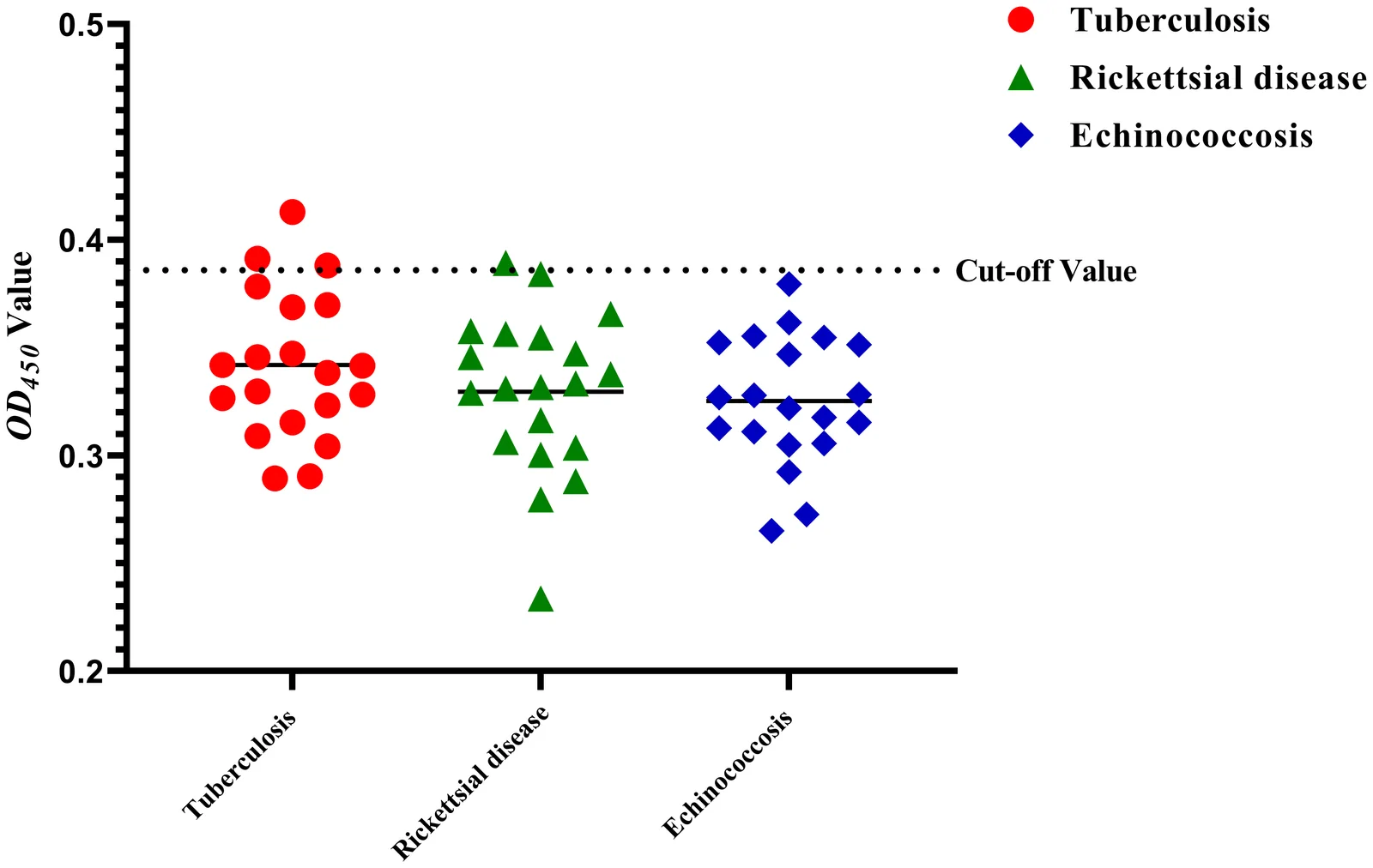
Specificity evaluation of the rSOD indirect ELISA against common endemic infectious diseases in the Xinjiang region. The scatter plot shows the OD_450_ values for serum samples from patients with tuberculosis (red circles), rickettsial disease (green triangles), and hepatic echinococcosis (blue diamonds). The horizontal red dotted line indicates the cut-off value (0.386). Cross-reactivity rates were 15% (3/20) for tuberculosis, 5% (1/20) for rickettsiosis, and 0% for echinococcosis.

A total of 60 serum samples were enrolled in this study, with 20 samples each from patients with tuberculosis, rickettsiosis and hepatic echinococcosis, all confirmed by clinical gold-standard diagnostics. All samples were assayed in triplicate, and the cutoff value of 0.386 determined in this study was adopted as the positive criterion. The test results are presented in **Table 7** and **Figure 6**.

**Table 7:** Scatter plot of OD values of ELISA test results for common infectious disease serum samples in the Xinjiang region.

Sample type	Sample size	Positive number	Cross-reaction rate (%)
Tuberculosis	20	3	15.0
Rickettsioses	20	1	5.0
Hepatic echinococcosis	20	0	0.0

The results showed that among the three highly endemic infectious diseases in Xinjiang, 3 tuberculosis serum samples tested positive, with a cross-reaction rate of 15%; 1 rickettsiosis serum sample was positive, yielding a cross-reaction rate of 5%; and no cross-reactivity was observed in hepatic echinococcosis serum samples.

In addition to the head-to-head clinical sample validation, we further compared the cross-reactivity profiles of the rSOD-ELISA and the LPS-ELISA using the 60 serum samples from patients with tuberculosis, rickettsiosis, and hepatic echinococcosis. The detailed comparison is shown in **Table 8**.

**Table 8:** Comparative cross-reactivity of rSOD-ELISA and LPS-ELISA against endemic infectious diseases.

Sample type (n=20 each)	rSOD-ELISA Positive (Cross-reactivity rate)	LPS-ELISA Positive (Cross-reactivity rate)
Tuberculosis	3 (15%)	7 (35%)
Rickettsiosis	1 (5%)	5 (25%)
Hepatic echinococcosis	0 (0%)	2 (10%)

#### 3.5.3 Sensitivity test

**Figure 7:**
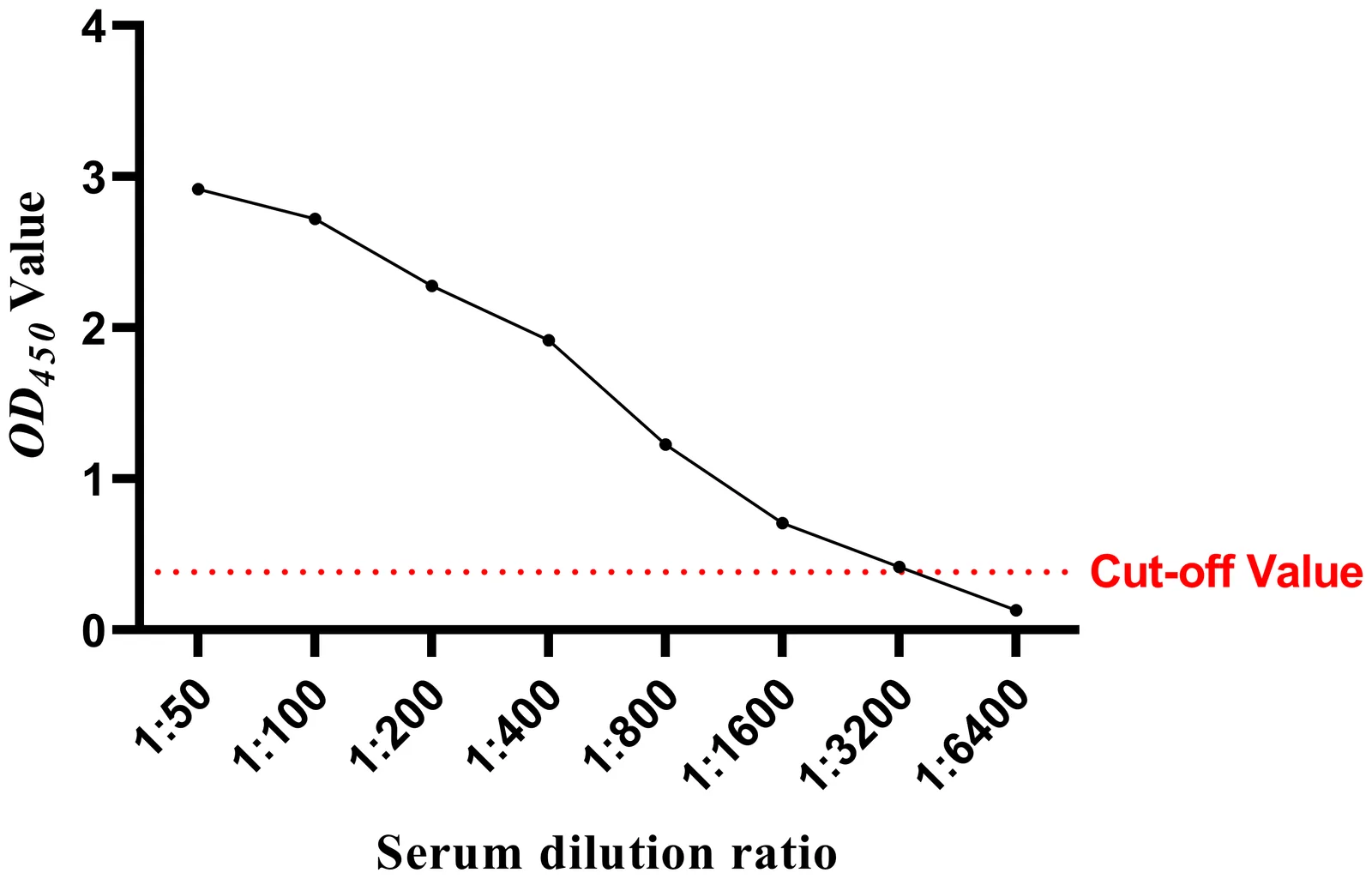
Sensitivity validation of the rSOD indirect ELISA using serial dilutions of confirmed positive serum. The curve shows OD_450_ values plotted against serum dilutions from 1:50 to 1:6400. The red dotted line indicates the cut-off value (0.386). The assay could still detect positive signals at a dilution of 1:3200, demonstrating high analytical sensitivity.

The analytical sensitivity of the rSOD-ELISA was evaluated using one representative confirmed positive serum sample. This sample was serially diluted from 1:50 to 1:6400. As shown in **Figure 7**, the OD_450_ values decreased with increasing dilution; however, the detection result remained positive at a 1:3200 dilution, indicating that the established ELISA method has high analytical sensitivity.

#### 3.5.4 Repeatability experiments

Intra-assay and inter-assay repeatability tests were carried out on 6 known serum samples using the same batch and three different batches of microplate. The within-assay coefficient of variation ranged from 0.93% to 4.60%, which was less than 10% and within the acceptable range. The inter-assay coefficient of variation ranged from 1.09% to 12.88%, which was less than the quality control requirement of 15%. The experimental data showed that the established ELISA method showed good repeatability both within and between batches. (**Table 9**).

**Table 9:** Repeatability of experimental results.

Serum number	Intra-assay repeatability			Inter-assay repeatability		
	Average	SD	CV (%)	Average	SD	CV (%)
P_1_	2.163	0.031	1.43	2.157	0.047	2.18
P_2_	1.070	0.010	0.93	1.057	0.03	2.84
P_3_	0.391	0.018	4.60	0.396	0.021	5.30
N_1_	0.325	0.006	1.80	0.314	0.027	8.59
N_2_	0.170	0.004	2.35	0.163	0.021	12.88
N_3_	0.178	0.003	1.69	0.184	0.002	1.09

P, Positive sample; N, Negative sample.

### 3.6 Test results of clinical samples

**Figure 8:**
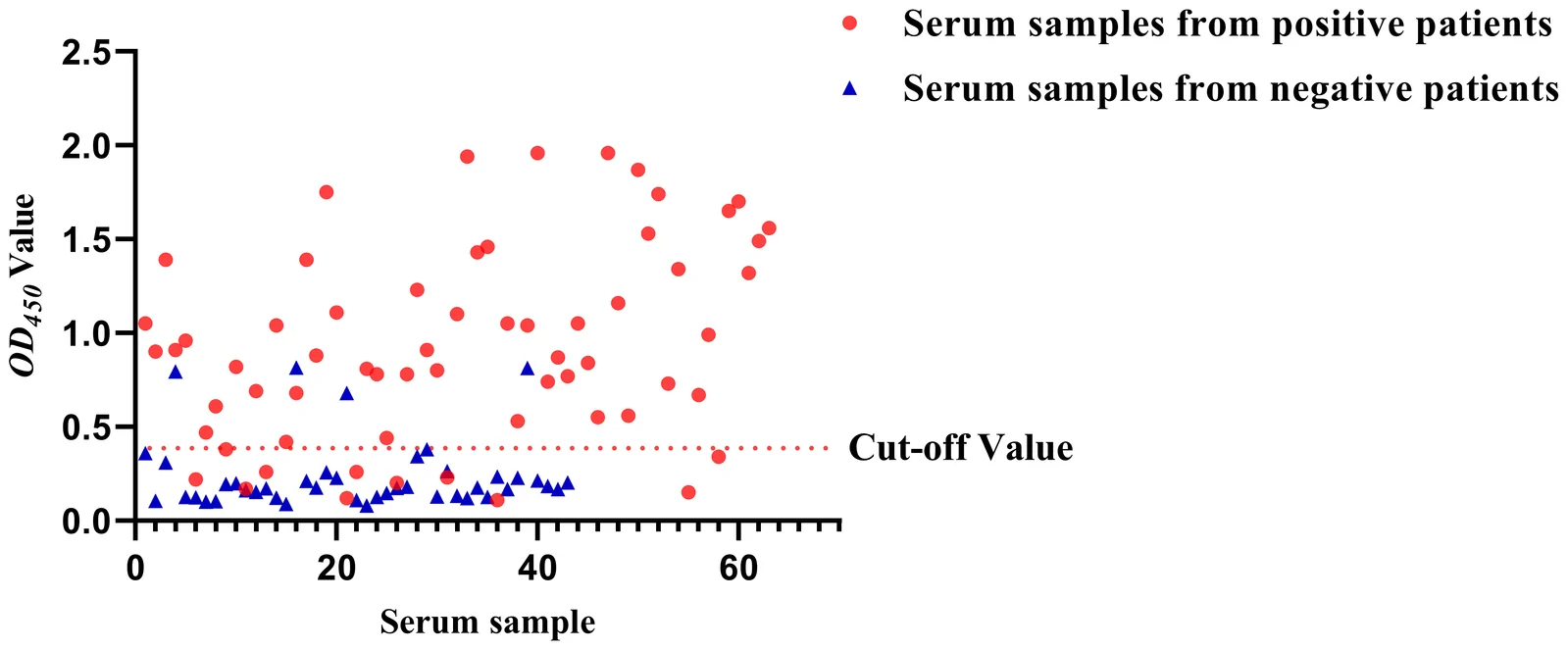
Clinical diagnostic evaluation of the rSOD indirect ELISA in 106 clinical samples. The scatter plot shows the distribution of OD_450_ values for serum samples from confirmed brucellosis patients (red circles) and healthy controls (blue triangles). The horizontal red dotted line represents the established cut-off value (0.386). With this threshold, the assay achieved a sensitivity of 82.5% and a specificity of 90.7%.

The indirect ELISA method was used to detect the serum of 63 brucellosis patients and 43 healthy people. With OD_450_=0.386 as the positive criterion, the positive rate of anti-SOD antibody was 82.5% (52/63) in the patient group and 9.3% (4/43) in the healthy control group (**Figure 8**). The difference in positive rate between the two groups was statistically significant (χ²=55.02, P<0.001), and the odds ratio (OR) was 46.09 (95%CI: 13.64-155.72). At this cut-off value, the sensitivity was 82.5% (95%CI: 71.4%-89.9%), the specificity was 90.7% (95%CI: 78.4%-96.3%), and the positive predictive value was 92.9% (95%CI: 78.4%-96.3%). 83.2%-97.5%) and negative predictive value 78.0% (95%CI: 64.8%-87.3%).

To provide a comprehensive overview of the diagnostic performance and the underlying gold standard components, the detailed cross-tabulation of the rSOD-ELISA results against the composite clinical diagnosis, as well as the individual SAT and culture results, is presented in **Supplementary Table 2**.

### 3.7 Comparison results with the LPS-ELISA kit

A total of 106 serum samples (63 brucellosis patients and 43 healthy controls) were detected by the LPS-ELISA method as a reference. Taking the clinical diagnosis results as the gold standard, the diagnostic sensitivity and specificity of the reference method were 80.9% (51/63) and 83.7% (36/43), respectively. The false positive rate of the healthy population was 16.3% (7/43). Compared with rSOD-ELISA established in this study, the sensitivity of the two methods was similar (82.5% vs 80.9%), while rSOD-ELISA was more specific and had a lower false positive rate (9.3% vs 16.3%) in healthy people. Although the false positive rate of rSOD-ELISA tended to decrease, this difference did not reach statistical significance (P = 0.248), possibly due to the relatively small sample size of the healthy control group (n=43).

The paired McNemar test with continuity correction was used to compare the difference in positive detection rate between the two methods. Among the 63 confirmed cases, there was one case with inconsistent results of the two methods (only rSOD-ELISA was positive, and LPS-ELISA was negative), and the test results showed that there was no significant difference in the positive detection ability of the two methods for brucellosis. Among the 43 healthy controls, there were 3 samples with inconsistent results (all were false positive by LPS-ELISA and negative by rSOD-ELISA), and the correction χ²=1.333, df=1, P = 0.248, indicating that there was no significant difference in the false positive rate between the two methods in the healthy population. However, rSOD-ELISA showed a trend of a lower false positive rate.

The consistency analysis showed that the overall coincidence rate of the two methods for all 106 samples was 96.2% (102/106). By Cohen’s Kappa test, the overall Kappa value was 0.92 (P < 0.001), indicating excellent concordance between the two methods. It should be emphasized that high agreement reflects the similarity of results, not diagnostic superiority. The independent advantage of the rSOD-ELISA was its greater specificity (90.7% vs. 83.7%) and lower false positive rate (9.3% vs. 16.3%), trends that suggest a clinically meaningful improvement with this kit.

## 4 Discussion

As an antioxidant, Cu/Zn SOD can protect *Brucella* from exogenous O2−, enabling it to survive in large numbers in macrophages and avoid oxidative killing mechanisms (Beck et al., 1990; Barquero-Calvo et al., 2007; Głowacka et al., 2018). When *Brucella* invades host macrophages, it will rapidly trigger the respiratory burst mediated by NADPH oxidase and produce a large number of reactive oxygen species (ROS) such as superoxide anion and hydrogen peroxide, which is the first line of defense of the innate immune system to clear intracellular pathogens. However, Cu/Zn SOD encoded by *B. melitensis* is mainly located in the pericytosolic space, which can quickly disintegrate O_2_^−^ into less toxic H_2_O_2_ and O_2_ before ROS is diffused into the bacterial cells, and then cooperate with catalase and alkyl hydroperoxide reductase of the bacteria to form a complete antioxidant network to completely relieve the oxidative killing effect of the host (Beck et al., 1990). This unique antioxidant capacity is the core virulence basis for *Brucella* to break through the host immune barrier and establish persistent intracellular infection (Tatum et al., 1992; Gee et al., 2005). More importantly, SOD was highly expressed throughout the whole *Brucella* infection cycle, and its antibody response appeared early and lasted for a long time. At the same time, SOD was positively correlated with bacterial load and infection activity (Tian et al., 2020), which provided a solid immunological basis for its use as a serological diagnostic antigen.

In this study, the prokaryotic expression vector of *Brucella* Cu/Zn SOD with an N-terminal signal peptide truncated was successfully constructed. Through codon optimization in *E. coli* and low-temperature induction at 25°C, the efficient soluble expression of recombinant protein was achieved. Compared with the previous scheme of obtaining SOD antigen by refolding of inclusion bodies in animal brucellosis studies, this method avoided the common loss of antigen activity during refolding, and laid the foundation for high stability and repeatability of the detection method. The indirect ELISA method using the recombinant protein as antigen was established. After system optimization and methodological verification, it showed good sensitivity (stable detection of 1:3200 diluted positive serum) and repeatability (intra-assay CV 0.93%-4.60%, inter-assay CV 1.09%-12.88%), which met the quality control requirements of clinical diagnostic reagents.

The results of clinical samples further confirmed the practical application value of this method. In the preliminary validation of 106 clinical samples in Xinjiang, the diagnostic sensitivity of the method was 82.5%, indicating that it had good clinical discrimination ability. It is worth noting that 9.3% (4/43) of brucellosis was positive in healthy controls, which was highly consistent with the epidemiological background of a high epidemic of brucellosis in Xinjiang. Studies have shown that the seropositive rate of the occupational population in Xinjiang is as high as 15%-20%, and some asymptomatic individuals may have inapparent infection or residual antibodies from previous infection. In this context, the choice of cutoff values is crucial to balance sensitivity and specificity.

The cutoff value (OD_450_= 0.386) was determined based on 40 clinically negative serum samples. Although the cutoff value (x+3SD) showed high sensitivity and specificity for the screening of clinical brucellosis samples, it showed high cross-reactivity with clinical tuberculosis samples. This phenomenon may be caused by a variety of factors. As a widely existing antioxidant enzyme, Cu/Zn SOD may have some conserved catalytic active center regions among different species. Although there are differences at the sequence level, the 3D structure of Cu/Zn SOD may retain some similarities. Previous studies have clearly reported that the serum of tuberculosis patients can cross-react with *Brucella* antigen. Some scholars have found that 9.2% of active tuberculosis patients have a positive reaction in anti-*Brucella* IgG ELISA, suggesting that tuberculosis infection itself can induce the production of cross-antibodies recognizing the *Brucella* SOD epitope (Piddington et al., 2001). In addition, Xinjiang is a double high incidence area of brucellosis and tuberculosis. According to the infectious disease detection of the Autonomous Region’s disease prevention and Control Bureau, tuberculosis and brucellosis ranked second and fifth in the number of reported cases in the Autonomous Region in January 2026. In this epidemiological context, some of the included tuberculosis patients may have mixed infection with asymptomatic or latent brucellosis, rather than simple serological cross-reaction.

The relatively elevated background OD in negative samples (mean = 0.263) may reflect both the high endemicity of brucellosis in Xinjiang and potential non-specific binding of the polyclonal secondary antibody. While the absolute OD values are higher than ideal, the P/N ratio of 4.06 and the high diagnostic accuracy validate the clinical utility of this cut-off. Future optimization could explore alternative blocking reagents or monoclonal secondary antibodies to further reduce background. Despite the slightly elevated background OD values observed in negative controls, the established cut-off value yielded a high specificity of 90.7%. This confirms that the rSOD-ELISA maintains robust discriminatory power. Compared to the conventional LPS-based ELISA, our rSOD-ELISA successfully reduced false positives in healthy controls from 16.3% to 9.3%. This improvement is particularly significant in Xinjiang, a region with high co-endemic rates of tuberculosis and brucellosis. The strong overall agreement (Kappa = 0.92) with the reference LPS kit further validates the reliability of rSOD-ELISA as a supplementary tool for human brucellosis screening in primary healthcare settings.

Crucially, when we further compared the cross-reactivity of the two ELISA methods against the endemic diseases, the commercial LPS-ELISA showed a noticeably higher rate of false-positive reactions (e.g., 7/20 in tuberculosis) compared to our rSOD-ELISA. This result underscores that rSOD-ELISA not only reduces false positives in healthy populations but also exhibits superior discriminatory capacity against clinically overlapping endemic infectious diseases.

In this study, rSOD-ELISA detected 11 false negative cases in 63 patients with confirmed brucellosis. In-depth analysis of this result is helpful to understand the applicable boundary of this method. The production of antibodies after *Brucella* infection has a clear time dependence: IgM antibodies usually appear 1 to 2 weeks after infection, while IgG antibodies need to reach detectable levels 2 to 4 weeks after infection (Smits et al., 2003; Mehrabani et al., 2015). Therefore, serum samples collected early in infection (within 2 weeks of illness onset) may be in the serologic window period, when specific IgG antibodies have not yet been fully produced, and any IgG-targeted ELISA method may give a negative result. In addition, the serum antibody level of brucellosis patients in the chronic phase was significantly lower than that in the acute phase, and IgG showed a continuous decline after the peak in the acute phase. Chronic patients in stable condition have low antibody levels and small fluctuations (Araj et al., 1986; Bai et al., 2023), and such samples can show negative results due to antibody concentrations below the detection limit of the method. The 11 false negative cases included in this study included both acute cases in the early stage of infection that had not completed serological conversion, and chronic cases with partial antibody level decline. The above two groups of people’s serological characteristics can reasonably explain the main causes of false negative results of this method. It also suggests that rSOD-ELISA has a higher sensitivity for the diagnosis of brucellosis in patients with a course of disease ≥3 weeks. For individuals suspected of early infection, chronic stable stage, or atypical humoral immune response, it is recommended to combine bacterial isolation and culture or molecular biological detection methods to improve the efficiency of pathogen detection and reduce the risk of missed diagnosis.

Although the false positive rate of rSOD-ELISA was lower than that of LPS-ELISA in healthy people (9.3%vs16.3%), the difference between the two groups did not reach statistical significance (P = 0.248). This result may be related to the limited sample size of healthy controls (n=43). Nevertheless, rSOD-ELISA successfully eliminated three cases of false positive reactions characteristic of LPS-ELISA (i.e., samples that were only positive for LPS-ELISA but negative for rSOD-ELISA), suggesting the potential advantage of SOD antigen in reducing non-specific cross-reactions.

This study provides the systematic evaluation of Cu/Zn SOD for human brucellosis serodiagnosis. However, several limitations should be acknowledged. Firstly, the relatively small number of clinical samples (n=106) in this single-center study may limit the generalizability of our findings. Secondly, the positive samples from healthy controls were not subjected to long-term follow-up, making it difficult to definitively distinguish between inapparent infections and residual antibodies from previous exposure. Future large-scale, multicenter prospective studies are warranted to further validate the clinical utility of rSOD-ELISA and to explore its potential for monitoring therapeutic responses in treated patients.

In conclusion, this study successfully established an indirect ELISA method based on rSOD of *Brucella* with high specificity, good sensitivity and repeatability, which was suitable for the epidemiological characteristics of brucellosis. This technique is expected to be a novel, reliable, and low cross-reactive serological diagnostic tool for brucellosis.

## Data Availability

The original contributions presented in the study are included in the article/**Supplementary Material**. Further inquiries can be directed to the corresponding author.
