# Supplementary material for: To establish an indirect ELISA method for the detection of human brucellosis based on signal peptide-truncated recombinant Cu/Zn SOD and its preliminary clinical verification
Source: Front Cell Infect Microbiol. 2026 Sep 8;16:1933037. doi: 10.3389/fcimb.2026.1933037 (PMC13593423; doi:10.3389/fcimb.2026.1933037)
Supplement: Supplementary file 1 [file DataSheet1.pdf]

Supplementary Information: Original Gel/Blot Images

Title: To establish an indirect ELISA method for the detection of human brucellosis based on signal peptide truncated recombinant Cu/Zn SOD and its preliminary clinical verification

Content: This PDF contains the original, uncropped full-length images of all SDS-PAGE gels used in the main figures. Due to limited laboratory equipment, these images were captured using a mobile phone camera. The raw images have not been processed or altered in any way. All relevant molecular weight markers (M) and sample lanes are clearly labeled.

Figure 1B. Agarose gel electrophoresis analysis of PCR products of colonies.

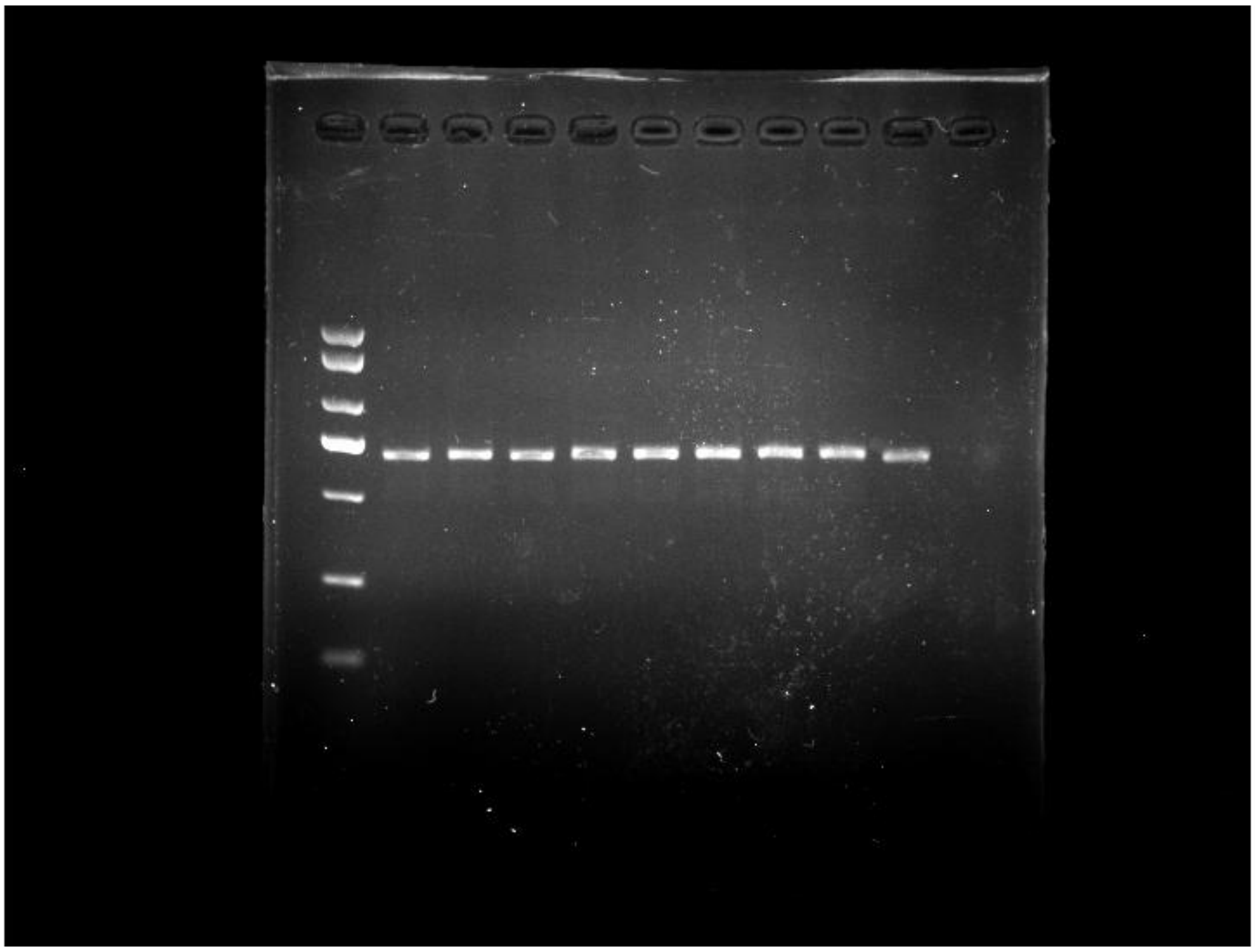

From left to right, they are:1. Marker;2-9. PCR product;10. Negative control

Figure 2A

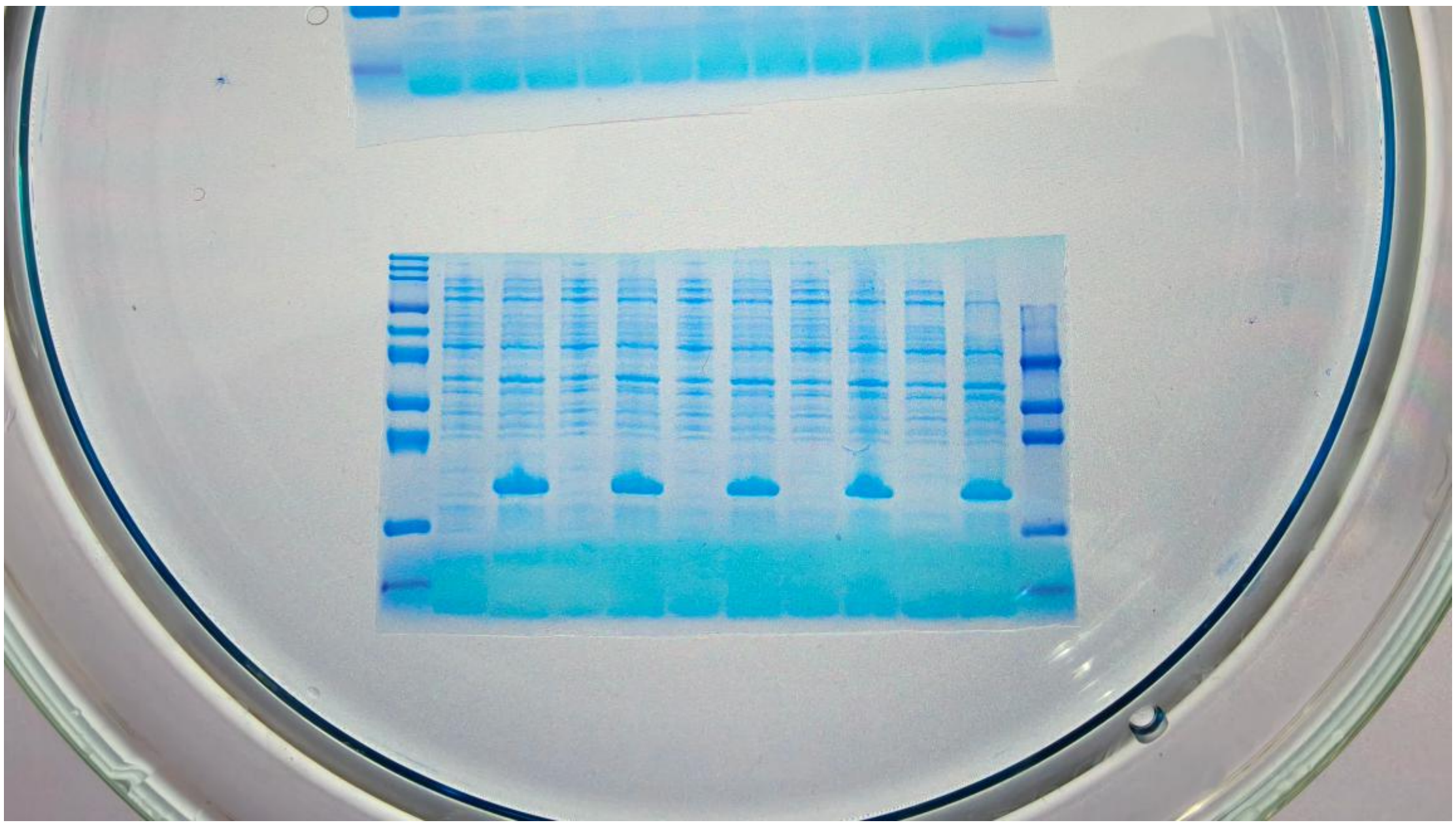

From left to right, the lanes are : Maker (8-195kDa); IPTG-induced BL21(DE3) competent cells were subjected to alternating loading of protein extracts before and after the induction process; Maker (10-180kDa)

Note: Image was captured by a mobile phone camera. The exposure and contrast have not been adjusted. The full membrane including all edges and visible marker bands is presented.

Figure 2B

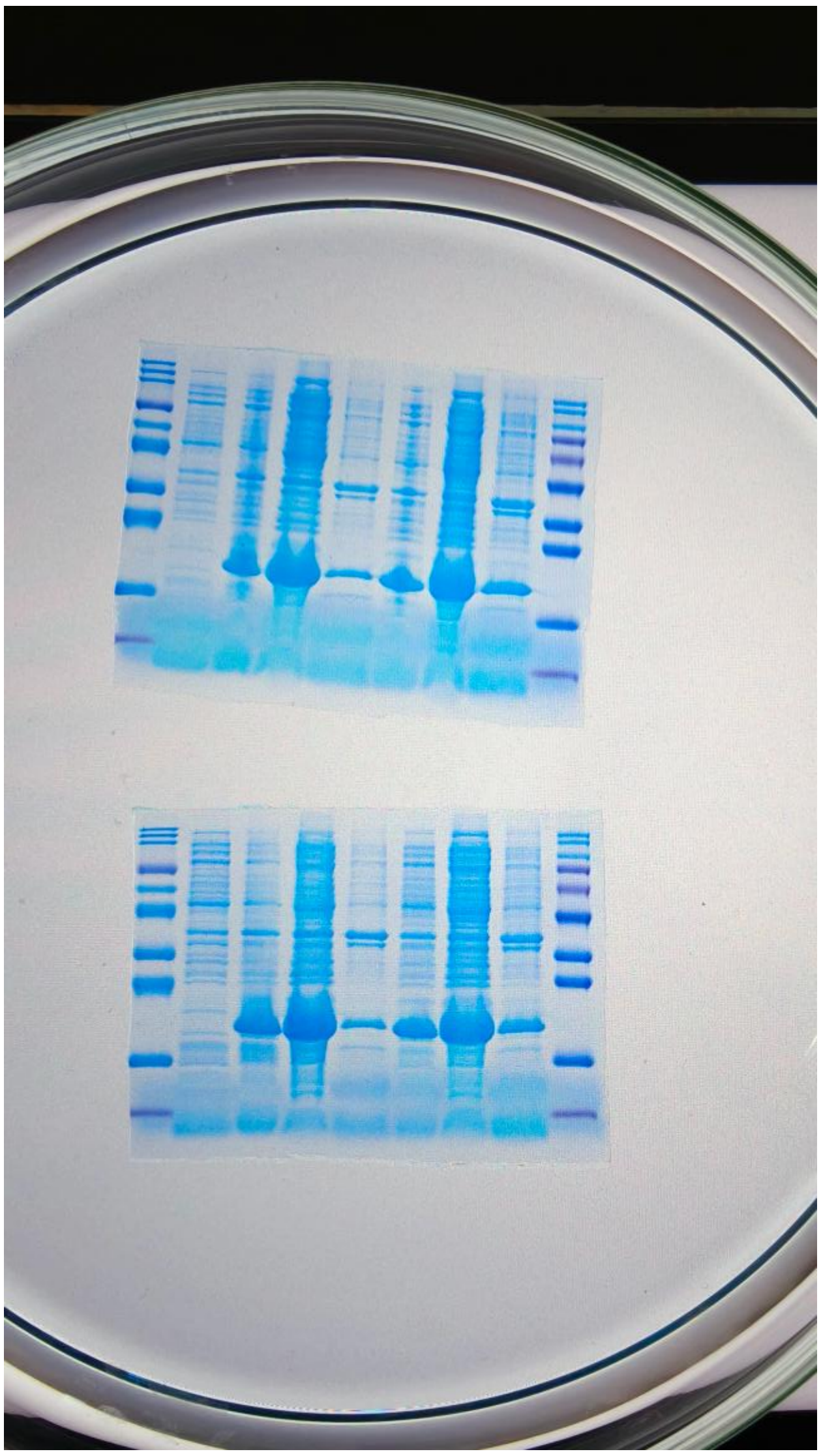

From left to right, the lanes are respectively: lane 1, protein marker (8–195 kDa); lane 2, uninduced BL21(DE3) whole-cell lysate (without IPTG); lane 3, whole-cell lysate after induction with IPTG at 37 °C; lane 4, supernatant fraction of the induced culture at 37 °C; lane 5, pellet (insoluble) fraction at 37 °C; lane 6, whole-cell lysate after induction at 25 °C; lane 7, supernatant fraction at 25 °C; lane 8, pellet fraction at 25 °C; lane 9, protein marker (10–180 kDa).

Note: Image was captured by a mobile phone camera. The exposure and contrast have not been adjusted. The full membrane including all edges and visible marker bands is presented.

Figure 2C

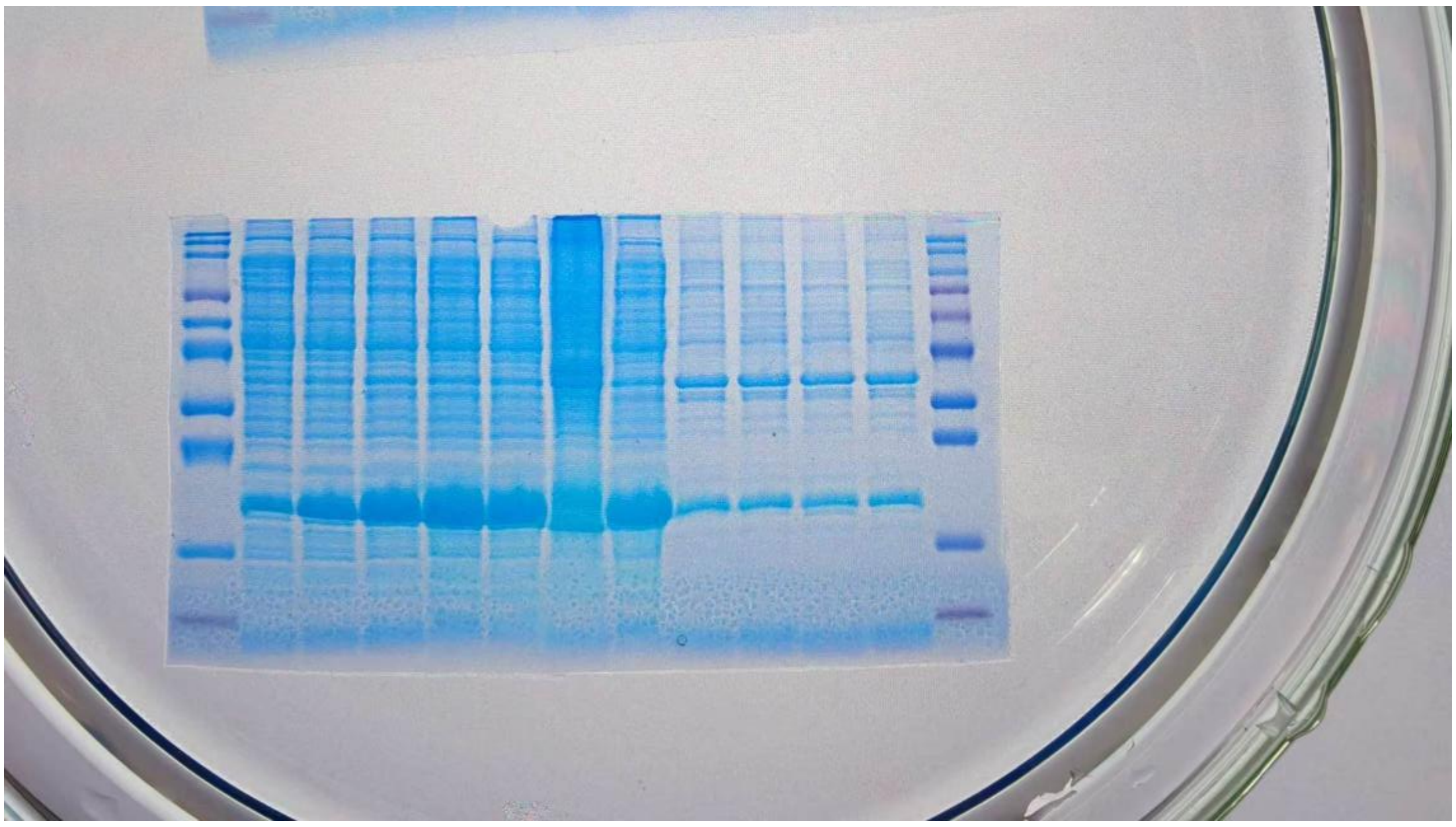

From left to right, the lanes are respectively: Lane 1, protein marker (8–195 kDa);

Lanes 2–8, supernatant fractions of BL21(DE3) cultures induced with IPTG at concentrations of 0.01, 0.02, 0.05, 0.1, 0.2, 0.5, and 1.0 mM, respectively, at 25 °C;

Lanes 9–12, not used in the main figure;

Lane 13, protein marker (10–180 kDa).

Note: Image was captured by a mobile phone camera. The exposure and contrast have not been adjusted. The full membrane including all edges and visible marker bands is presented.

Figure 2D

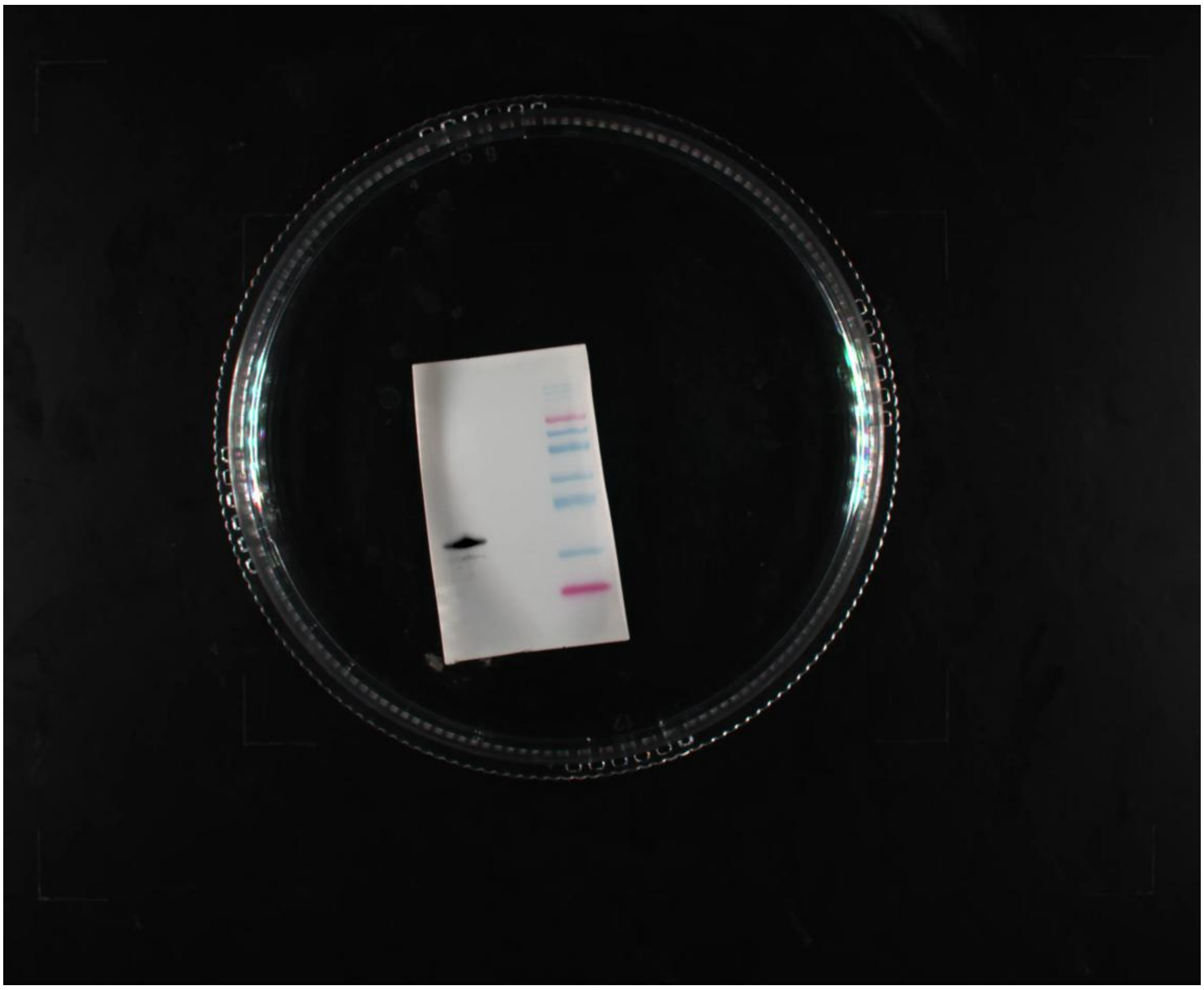

The expression of the target protein was detected by Western blot. The image shows the composite superimposition of the chemiluminescent signal and the pre-stained protein markers. From left to right: The first lane is the total cell lysate of cells induced (containing IPTG); the second lane is the total cell lysate of uninduced cells (without IPTG); the third lane is the protein molecular weight marker (8–195 kDa).

Figure 3

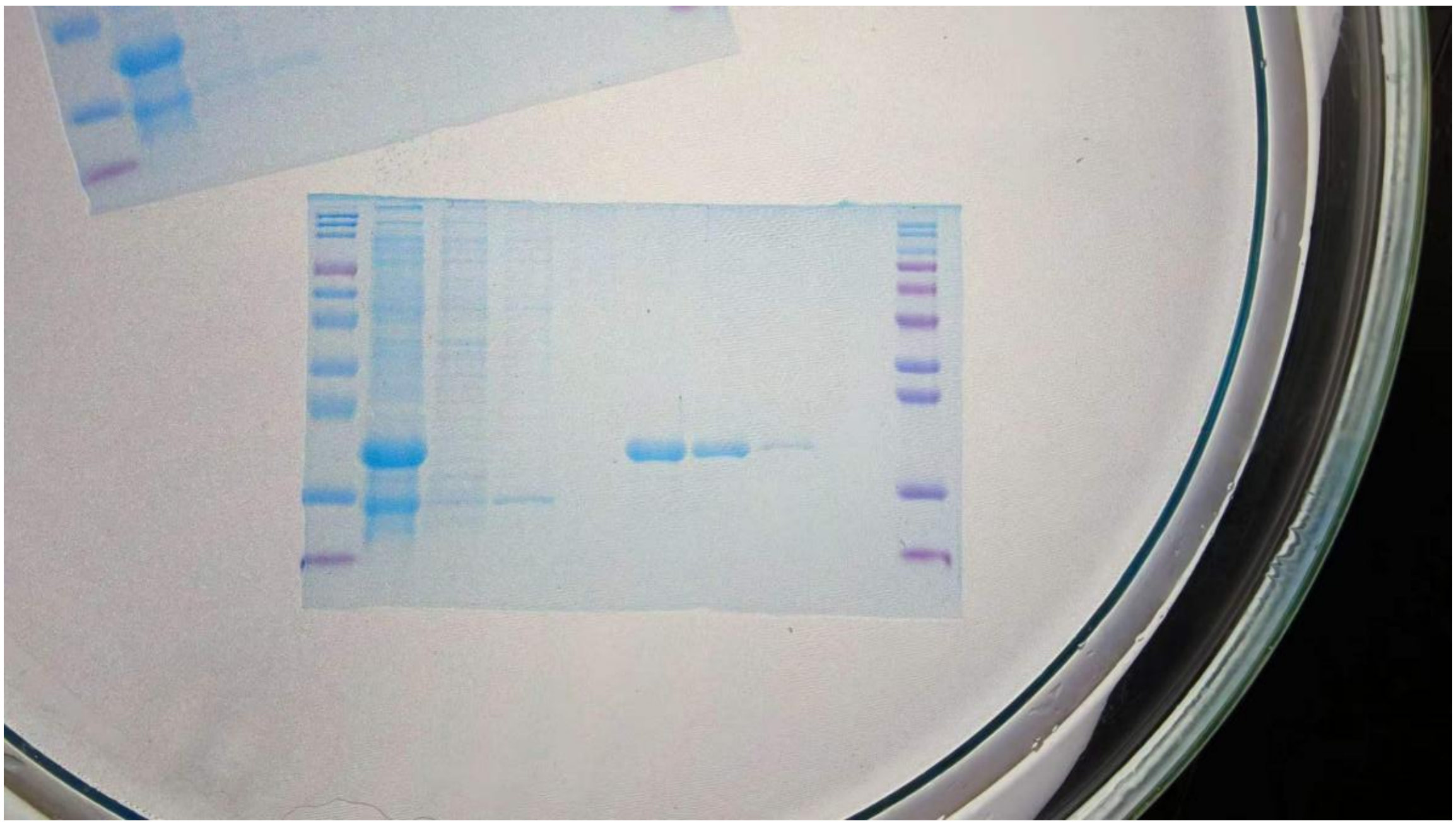

SDS-PAGE analysis of recombinant protein purification by Ni-NTA affinity chromatography. The gel image is shown in its full-length, unprocessed form. Lanes from left to right are as follows:

Lane 1, prestained protein marker (8–195 kDa);

Lane 2, soluble supernatant of recombinant BL21(DE3) cell lysate;

Lane 3, flow-through fraction;

Lane 4, wash fraction with 20 mM imidazole (Wash 1);

Lane 5, wash fraction with 50 mM imidazole (Wash 2);

Lanes 6–9, elution fractions with 250, 300, 400, and 500 mM imidazole, respectively;

Lane 10, prestained protein marker (10–180 kDa).

All fractions were resolved by SDS-PAGE and visualized by Coomassie Blue staining.

Note: Image was captured by a mobile phone camera. The exposure and contrast have not been adjusted. The full membrane including all edges and visible marker bands is presented.
