# Supplementary material for: To establish an indirect ELISA method for the detection of human brucellosis based on signal peptide-truncated recombinant Cu/Zn SOD and its preliminary clinical verification
Source: Front Cell Infect Microbiol. 2026 Sep 8;16:1933037. doi: 10.3389/fcimb.2026.1933037 (PMC13593423; doi:10.3389/fcimb.2026.1933037)
Supplement: Supplementary file 2 [file DataSheet2.pdf]

Supplementary Data S1. Details of the Cohen's Kappa coefficient calculation.

1. Cross-tabulation of rSOD-ELISA and LPS-ELISA results (n = 106)

	LPS-ELISA Positive	LPS-ELISA Negative	Total
rSOD-ELISA Positive	A = 55	B = 1	56
rSOD-ELISA Negative	C = 3	D = 47	50
Total	58	48	N = 106

2. Calculation steps

Observed agreement (Po):

$$Po = \frac{A + D}{N} = \frac{55 + 47}{106} = \frac{102}{106} \approx 0.9623$$

Expected agreement based on chance (Pe):

$$Pe = \frac{[(A + B) + (A + C) + (C + D) + (B + D)]}{N^2} = \frac{[(56 \times 58) + (50 \times 48)]}{106^2} \approx 0.5027$$

Cohen's Kappa coefficient (κ):

$$\kappa = \frac{Po - Pe}{1 - Pe} = \frac{(0.9623 - 0.5027)}{1 - 0.5027} = \frac{0.4596}{0.4973} \approx 0.92$$

3. Interpretation

According to the guidelines by Landis and Koch (1977), a Kappa value of 0.81–1.00 indicates "almost perfect agreement". The calculated κ value of 0.92 ($P < 0.001$) demonstrates strong consistency between the established rSOD-ELISA and the reference LPS-ELISA.

Supplementary Table S2. Cross-tabulation of rSOD-ELISA results with the gold standard clinical diagnosis and individual testing methods.

Table A. Overall diagnostic performance of rSOD-ELISA compared to the composite gold standard (clinical diagnosis based on SAT and/or culture).

rSOD-ELISA	Clinical gold standard (Confirmed patients)	Clinical Gold Standard (Healthy Control)	total
Positive	52 (TP)	4 (FP)	56
Negative	11 (FN)	39 (TN)	50
Total	63	43	106

Note: Clinical diagnosis (n=63) was established based on the 2023 National Protocol for Brucellosis Diagnosis and Treatment, requiring positivity in either SAT or bacterial culture. SAT: Tube Agglutination Test; TP: True Positive; FP: False Positive; FN: False Negative; TN: True Negative.

Table B. Results of rSOD-ELISA in SAT-positive and Culture-positive patient subgroups (n=63).

Patient Group	Number of patients (n)	rSOD-ELISA Positive	rSOD-ELISA Negative
SAT-positive*	59	49	6
Culture-positive*	17	13	3

Note: *These subgroups are not mutually exclusive; some patients were positive for both SAT and culture. The total number of unique patients is 63, with an overall rSOD-ELISA sensitivity of 82.5% (52/63).
